# Stem cell–based therapies in pediatric disorders: translational advances, unresolved challenges, and future horizons

**DOI:** 10.1186/s13287-026-05204-0

**Published:** 2026-08-15

**Authors:** Hany E. Marei

**Affiliations:** Department of Cytology and Histology, Faculty of Veterinary Medicine, Mansoura University, Mansoura, 35116 Egypt

**Keywords:** Pediatric stem cell therapy, Regenerative medicine, Mesenchymal stem cells (MSCs), Induced pluripotent stem cells (iPSCs), Neural stem cells (NSCs), Clinical translation, Extracellular vesicles (EVs), Cell-based therapy, Gene editing, Regulatory challenges

## Abstract

Pediatric disorders consist of genetic, hematologic, neurologic, autoimmune, and inflammatory diseases. These conditions impose long-term health challenges on children, despite many advancements with conventional medicine. Although conventional treatments increase life expectancy and provide better disease control, many present challenges such as toxicity, insufficient control of the disease, and adverse effects on normal growth, development, and quality of life. Many researchers have shown increased interest in using stem cell therapies as an alternative to current medications to allow for complete, sustainable repair of damaged tissues and modification of disease processes (i.e., using stem cells to regenerate tissue or change the way in which a disease occurs). This paper will provide the current information on stem cells used in the treatment of children and the many different types of stem cells, including: hematopoietic stem cells (and their derivatives), mesenchymal stem cells (and their derivatives), induced pluripotent stem cells, embryonic stem cells, tissue-specific progenitor cells, extracellular vesicles, and bioengineered products. This paper will also discuss what is known about the stem cells listed as well as their methods of action, where they might currently be better utilized, and future uses of these cells in children for a variety of types of pediatric diseases. Because each stem cell type listed has very different scientific background and clinical evidence, there is much variability in the amount of scientific evidence available to support stem cell therapies. For example, hematopoietic stem cell transplantation (HSCT) has over 50 years of clinical experience; thus, there are many studies defining the clinical efficacy and long-term outcomes associated with HSCT. Conversely, while there are many published studies supporting the use of mesenchymal stem cells (MSCs), extracellular vesicles (EVs), gene-edited cells, organoids, and many induced pluripotent stem cell-derived therapies, more evidence (clinical and basic science) is still needed to fully establish efficacy for the use of these various stem cells in children with pediatric diseases. In addition to needing more clinical evidence, stem cell-based therapies face many important challenges to the advancement of these therapies, including (but not limited to) long-term safety assessments, manufacturing standardization, regulatory oversight, ethical concerns, and equitable access to advanced therapies. Addressing these challenges will be important for future advances in the use of regenerative medicine in pediatric patients which will also require the rigorous evaluation of new stem cell therapies, the continued improvement of translational mechanisms, and the ongoing incorporation of new techniques (e.g., genome editing, organoid modeling, EV therapeutics, bioengineering, and artificial intelligence) to advance regenerative medicine and demonstrate its value through safe and reproducible clinical trial results.

## Introduction

### The worldwide impact of childhood illnesses

Global health will be affected by diseases that occur throughout childhood and by environmental factors; however, there will also be long-term consequences of such early-life health issues. Diseases that affect children can include infection, genetic disorders, cancer, and immune deficiencies. A lot of children who are ill are in critical stages of their growth and development because many organ systems are not yet mature and because there are usually only small reserves of energy in the body during these times. Illness in early life can have lasting effects on a child’s physical growth, cognitive development, and overall quality of life.

Infections and diseases that cause immune system deficiencies will continue to significantly affect the health of children all over the world. For example, children who have received a stem cell transplant are very likely to experience opportunistic and invasive fungal infections due to their inability to fight off infections, and also because their immune system is still trying to recover from its previous destruction due to disease treatment, especially in countries where the health system is not developed or equipped to meet children’s health care needs. Although there are millions of children worldwide who suffer from diseases such as sickle cell anemia, which causes extreme amounts of recurrent pain, acute complications, progressive damage to the organs, and impaired development, these examples show that there is potential for a child to develop lifelong problems due to childhood disease [1, 2].

Another major concern in the world regarding health is childhood and adolescent cancer. While the number of children surviving has increased in the last few decades, many will experience long-term health problems such as heart-related problems, diabetes, reduced physical function, constant pain, and limitations in their ability to function. Therefore, pediatric oncologists have spent quite a bit of time evaluating ways to provide cancer therapy that is effective based on what they know, and bring those therapies to today’s children; however, they have also made it a goal to reduce the number of “bad” effects of the therapies and increase the quality of life of their patients after therapy [3, 4].

There are still very few treatment options available for children with serious, chronic diseases, and include illnesses such as necrotizing enterocolitis or certain types of interstitial lung disease (although rare), which are some of the leading causes of morbidity and mortality in those affected by them and therefore require specialized care and diagnosis of those who develop these rare diseases during early life. Similarly, congenital and early-onset immunodeficiency diseases require complex evaluations to diagnose and treat children who develop those diseases. It is clear that these disorders impose a high cost on families and the health care system and highlight the importance of more accurately identifying how to provide durable and appropriate treatments for children suffering from serious, chronic diseases [5, 6].

### Constraints of existing treatment methodologies

Despite major advances in pediatric medicine, many diseases still pose significant therapeutic challenges due to the clinical, biological, and technical limitations of current therapies. Among hematologic and oncologic diseases, both intensive chemotherapy and hematopoietic cell transplantation (HCT) are often necessary to achieve disease control. Although these interventions may save a child’s life, they are often associated with significant risk to the child, including but not limited to organ toxicity, inhibited growth or development, and most importantly long-term health complications.

When using allogeneic HCT to treat a pediatric patient, there is also a limitation imposed by the requirement for immunosuppression to prevent graft-versus-host disease, which creates a very narrow therapeutic range. The increased immunosuppression also increases the risk of developing opportunistic infections, and invasive fungal infections continue to be an important source of morbidity and mortality for pediatric transplant recipients. All of these issues demonstrate inadequacies in current treatment paradigms and emphasize the need for greater focus on more effective, less toxic Treatment Strategies [1].

GVHD and other chronic consequences make even “curative” transplantation difficult [6]. Most current approaches to modulating the immune response (immunomodulatory strategies) aim to manage alloreactivity and sustain protective immunity. However, achieving this has been limited, and preserving both immune functions while inducing immunosuppression remains challenging. Because of the way in which graft-versus-host disease is treated or prevented (often through prolonged use of immune suppression), hosts may be susceptible to infections/lowered immunity during periods of prolonged suppression of immune activity.

Therapeutic approaches in pediatric oncology are subject to similar limitations. Although advances in both the treatment regimen and refinement of protocols have improved the survival of children with cancer, the relative increases in survival have been accompanied by a growing appreciation for the long-term toxicities associated with the use of cytotoxic (non-targeted, non-selective) agents. As a result of this non-selective nature of cytotoxic agents, those who are cured of their malignancy (i.e., survivors) will likely be at a higher risk for developing severe cardiometabolic, aerobic capacity-reducing, endocrine dysfunctions, and secondary malignancies over the course of their life. These late effects underscore the need for targeted therapeutic agents that achieve efficacy while minimizing long-term toxicity/side effects [4]. Supportive care measures, especially those that reduce acute and chronic treatment-related toxicities, are insufficient to reduce cytotoxic therapy’s physiological effects [5].

One of the challenges faced in pediatric oncology is balancing the effectiveness of treatment with the potential for long-term toxicities. Even though patients are now living much longer because of advances in treatment, many of those survivors encounter complications such as cardiometabolic, endocrine, neurocognitive and musculoskeletal aftereffects from their treatment. This compelling need for improved outcomes continues to fuel interest in developing targeted, biologically-based therapies to minimize the risk for these aftereffects.

In addition, there are even fewer treatment options for noncancerous conditions than there are for cancers. The mechanism of action (how a medicine works) and the incompleteness of knowledge of how the disease develops limit researchers’ ability to design and develop ways to create specific treatment(s) for the disease based upon their understanding of how the disease develops (pathophysiology). For example, in neonatal inflammatory diseases such as necrotizing enterocolitis, there are no specific treatments available that target the underlying disease. Therefore, treatment remains largely a means of providing comfort until the patient recovers. These observations illustrate the broader problem of translating potentially effective methods identified in preclinical research studies into safe, effective, and clinically useful therapies for pediatric patients [7]. Rare genetic immunological illnesses are more difficult to treat because many regions lack diagnostic, testing, and treatment capacity [8].

Current therapeutic techniques in pediatric medicine do not account for sufficient biological relationships or flexibility to address the diverse range of pediatric illnesses and disease processes across development. Existing treatments focus on symptom management or on eliminating pathogenic cells. Consequently, physicians continue to find that they must tradeoff between achieving sufficient control over a child’s illness and limiting side effects from the therapy.

This highlights the need for more mechanistically informed and biologically targeted methods to treat these conditions. Developing improved disease models through technologically enhanced in vitro (laboratory) and in vivo (animal) studies will ultimately help researchers better understand the pathophysiology of the pediatric population, thereby enabling safer and more selective interventions. Interventions must be directed toward the biology of the disease rather than relying on broad cytotoxic or nonspecific modes of action to limit collateral tissue injury while maintaining high therapeutic efficacy [10].

### The potential of stem cell–based therapies in pediatric populations

Traditional kinds of treatment are often limited by toxicities, a general lack of specificity, and the requirement for long-term administration and support. This has generated considerable interest in researching alternative stem cell-based treatments for children with diseases. Certain types of stem cells, particularly neural stem cells, have been shown to migrate to areas surrounding injury in the central nervous system and to deliver therapeutic agents to those target areas. More generally, stem cells have the potential to facilitate tissue repair, modulate immune responses, and deliver targeted substances capable of biological effects (i.e., bioactive factors), making them well-suited for the treatment of disorders common in early life with defective repair processes.

Hematopoietic cell transplantation (HCT) is the most common form of stem cell-based therapy in children and has been used to treat multiple types of malignant and non-malignant diseases. Because HCT can restore the hematopoietic and immune systems after the disease has been treated, it is associated with high-risk complications such as graft vs. host disease, and patients who undergo HCT and have a depressed immune system are particularly susceptible to life-threatening fungal infections. Given the substantial limitations of this model, there is a strong need for additional stem cell-based therapies that can enhance patients’ immune reconstitution while minimizing complications associated with HCT [9, 10].

Chronic complications, including graft-versus-host disease (GVHD), are significant goals for improved stem cell technology with known immunomodulatory characteristics. Standard immunosuppressive agents typically provide inadequate protection and can also impair the host’s ability to defend itself. Conversely, appropriately characterized engineered or selected stem cell populations have the potential to contribute to immune regulation (i.e., enhancing tissue integrity) and to immune reconstitution in pediatric patients at risk [11, 12].

Stem cells may play a role in the future treatment of complications that arise during remission from childhood cancers. Improvements in treatment options have led to increased survival rates among patients with childhood cancer. A significant number of these patients will go on to develop long-term illnesses caused by their cancer treatment, such as nutritional deficiencies, heart problems, and decreased ability to be physically active as a result of their treatment with chemotherapeutic agents. Researchers are beginning to investigate whether the use of regenerative cells can improve patients’ function and restore organ or tissue damage resulting from treatment when other forms of medical treatment are ineffective [13].

In addition to treating diseases directly, stem-cell based treatments can aid tissue protection through immunomodulation, enhancing the body’s own reparative mechanisms, and decreasing inflammatory damage. These attributes are very important in the treatment of children, where the main goal of therapy is to preserve the normal growth and development of organs. This can be achieved by regulating the inflammatory response in an orderly manner, activating endogenous repair pathways, and reducing or eliminating systemic exposure to cytotoxic agents. The combined approach of providing tissue protection and specifically modulating injury responses may reduce collateral damage and enhance functional recovery in pediatric patients undergoing definitive therapy [14, 15].

Therapies using stem cells can deliver biologically active factors to injured sites, facilitating tissue repair and functional recovery; however, their mechanisms differ between malignant and non-malignant pediatric disorders. In malignant disorders, the goal is eradication; while in non-malignant disorders, the goal is restoration. Preclinical data suggest that mesenchymal stromal cells (MSCs) may improve repair of epithelial tissue (e.g., in necrotizing enterocolitis) by inhibiting excessive inflammation and aiding the restoration of barrier function impaired by conventional pharmacological treatments. Despite intermittent success in translating these findings into approved therapies, barriers remain, including challenges with scale and standardization of manufacturing, delivery optimization, and demonstrating consistent clinical efficacy [16, 17].

Pediatric patients with congenital immunodeficiency resulting from abnormal developmental pathways may be good candidates for stem cell repair approaches aimed at restoring functional immune systems. Concerns about genetic modification, cellular instability, variability in manufacturing, and the ability to deliver stem cells to affected tissues have limited the clinical application of these approaches. Before these techniques can be widely used in pediatrics, it is important to ensure cell viability and safety, as well as prolonged engraftment [18, 19].

### Goals and limits of this review

Although many findings remain early-stage, new research indicates that stem cell treatments can alter the biology of diseases affecting our children. In addition to treating the underlying medical condition, many of these treatments exhibit functional versatility, including modulating inflammation, enabling immune system regeneration after destruction, and repairing damaged tissues in a child’s body. As stem cell technologies (i.e., engineering, gene editing, and disease modeling) advance in precision and reliability, stem cell-based treatment approaches are moving out of “experimental” phases and into “carefully regulated” phases for clinical application in select pediatric conditions [9, 10].

Additionally, this review will evaluate whether or not the use of stem cell-derived platforms could address long-term complications related to nutritional dysfunction, cardiometabolic dysfunction, and decreased physical capacity in pediatric cancer survivors. Furthermore, emerging evidence indicates that mesenchymal stromal cells protect tissues and reduce inflammation associated with conditions such as necrotizing enterocolitis [5, 16].

Potential applications of stem cell therapy for immune disorders include the use of genetically modified HSCs to treat genetic immunodeficiencies and mesenchymal stem cells to treat GVHD. These examples demonstrate the potential immunomodulatory and restorative abilities of stem cell-based therapies; however, many questions remain regarding stem cells’ capacity to migrate, persist, engraft long-term, and integrate functionally into a predominantly developing pediatric environment with variable regenerative capacity. Lack of knowledge in these areas makes clinical application difficult, and further research is needed to elucidate the mechanisms of action [9].

The long-term safety of stem cell therapy for children has not been thoroughly researched. Possible risks may include abnormal differentiation/cell fate, unwanted immune responses, the development of cancer or tumors, or any unexpected changes due to the surrounding environment. In addition, multiple preclinical models are available, indicating that results will vary depending on the type of stem cell used. Therefore, many complicating issues should be carefully considered before the general use of these therapies in patients [1, 3].

The lack of consistent outcomes from regenerative treatments indicates that we need to thoughtfully integrate regenerative techniques into the established standard of care. We will consider using stem cell treatments as adjunctive therapies to optimize supportive strategies, such as nutrition and cytoprotective therapies, thereby improving their effectiveness while reducing the risk of complications [4, 20] Therapies using stem cells may enhance tissue repair and modulate inflammation. However, they represent only one of many therapies that can be used for this purpose; others include improved conditioning regimens, targeted immune-modulating drugs, and structured rehabilitation programs. All of these approaches will provide the greatest clinical benefit with the least harm [14, 15].

This review summarizes the current state of stem cell use in pediatric treatment, evaluates how pediatric stem cell therapies might be applied to life-threatening conditions, and identifies limitations to their widespread clinical use. Special attention is paid to the distinction between preclinical and clinical studies, as well as to potential research strategies to support the safe and effective use of stem cells in the treatment of children with serious illnesses. This review is organized using a translational approach, grouping evidence by cell type or disease category. Unlike most narrative review pieces that summarize stem cell therapy by cell type or disease category, this review places particular emphasis on distinguishing clinically established therapies from those under investigation or in clinical trials. In the context of developing an assessment of the current state and future trajectory for stem cell therapies, important components of this assessment include a critical analysis of the available evidence with regard to quality and inherent limitations, identification of barriers to clinical application of stem cell therapies, and consideration of unique biological traits found in children that may affect therapeutic outcome. This review is structured to include scientific, clinical, ethical, regulatory, and manufacturing perspectives to provide a balanced assessment of the current and future state of stem cell therapies in pediatric medicine.

## The function of types of stem cells in pediatric medicine

**Fig. 1:**
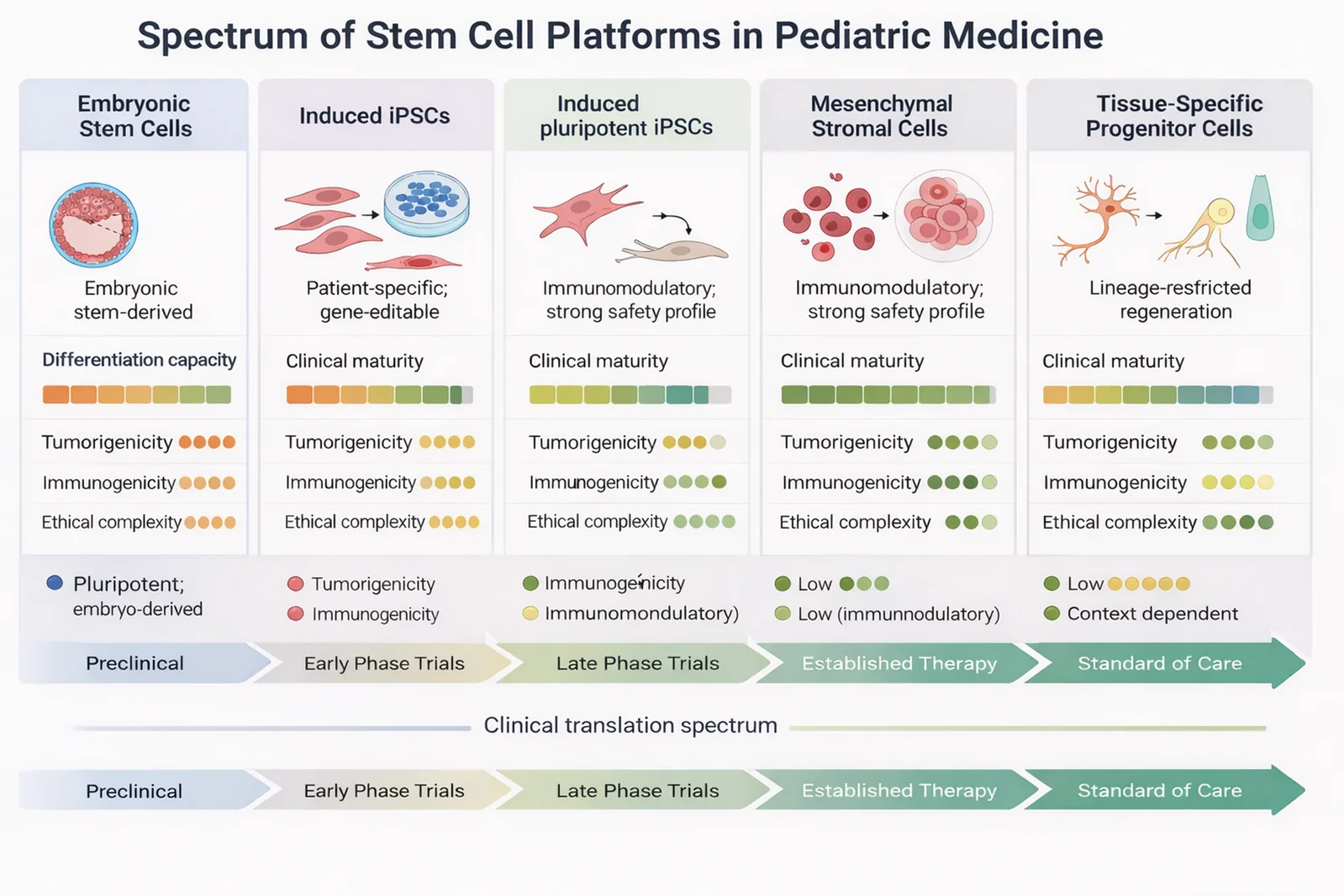
Spectrum of Stem Cell Platforms in Pediatric Medicine. The principal stem cell platforms for pediatric regenerative medicine are depicted. Embryonic stem cells (ESCs) have unlimited pluripotentiality but raise ethical concerns and pose a greater risk of tumor development. Induced pluripotent stem cells (iPSCs) have the potential to develop patient-specific and gene-modified therapies; however, their safety and genetic stability have not yet been established. Mesenchymal stromal cells (MSCs) possess strong immunomodulatory properties and have been shown to be safe for pediatric use. Hematopoietic stem cells (HSCs) are the best-studied and most clinically available stem cell product and are considered the standard of care for all pediatric hematological disorders requiring stem cell therapy. The various types of progenitor cells possess lineage-restricted regenerative capacity and carry a lower risk of cancer. The diagram illustrates the levels of differentiation ability, translational readiness, and regulatory hurdles across stem cell platforms. *This conceptual framework was adapted from previously published methodologies and synthesized based on insights from key studies in stem cell biology and pediatric regenerative medicine*,* including *[25, 103–107]

Stem cells exist in various forms and can be used to promote tissue repair and restore function in children with illnesses caused by multiple factors, including those related to organ development. Stem cells have diverse physiological characteristics and beneficial uses in patient treatment, but they also present varying degrees of safety, ethical, and legal implications. A more thorough discussion of these stem cell groups will follow in this article, along with their role in the treatment of pediatric diseases. Figure 1 shows the alignment of the main stem cell platforms onto a biological and translational continuum in pediatric medicine. Understanding the relative breadth of differentiation, clinical readiness, and safety associated with each cell type is crucial for matching the appropriate therapy to the biology of the disease, the developmental context, and the long-term expectations for the outcome.

### Embryonic stem cells and ethics

Preimplantation embryos develop inner cell masses that give rise to ESCs. These cells have the potential to differentiate into all somatic cell types and can therefore be used for disease modelling, drug discovery, and regenerative medicine research. The potential for creating ESCs, however, also raises significant ethical concerns regarding the storage and destruction of embryos used in ESC production, which will ultimately affect regulatory oversight and public policy, leading to national regulations on ESC research and limiting research and the clinical translatability of ESCs. In terms of using ESCs for any childhood disease, other factors must be considered in regard to possible tumor formation, immune response, and long-term effects of using ESCs. National regulations in many countries have limited researchers’ and clinicians’ ability to conduct their work, thereby slowing the translation of ESCs into clinical practice [21–23].

### Induced pluripotent stem cells: potential for the individual patient

The use of iPSC technology for personalized treatment in children is one of the most scientifically significant breakthroughs in regenerative medicine to date; however, the majority of proposed uses are still in preclinical development or early stages of clinical use, and problems remain unresolved regarding stability of the genomic information of these cells, complexity in their manufacture, risk of developing tumors from these cells, and approval by regulatory agencies.

The expansion of regenerative medicine has been enabled by induced pluripotent stem cells (iPSCs), which allow the generation of patient-specific pluripotent cells from adult somatic cells. This helps mitigate the ethical issues surrounding the use of embryonic cells while enabling individualized modeling approaches. In pediatrics, iPSC lines derived from children with genetic disorders have been used to study disease mechanisms, identify potential treatment candidates, and evaluate whether autologous cell therapy could be used in patients [17, 24].

**Fig. 2:**
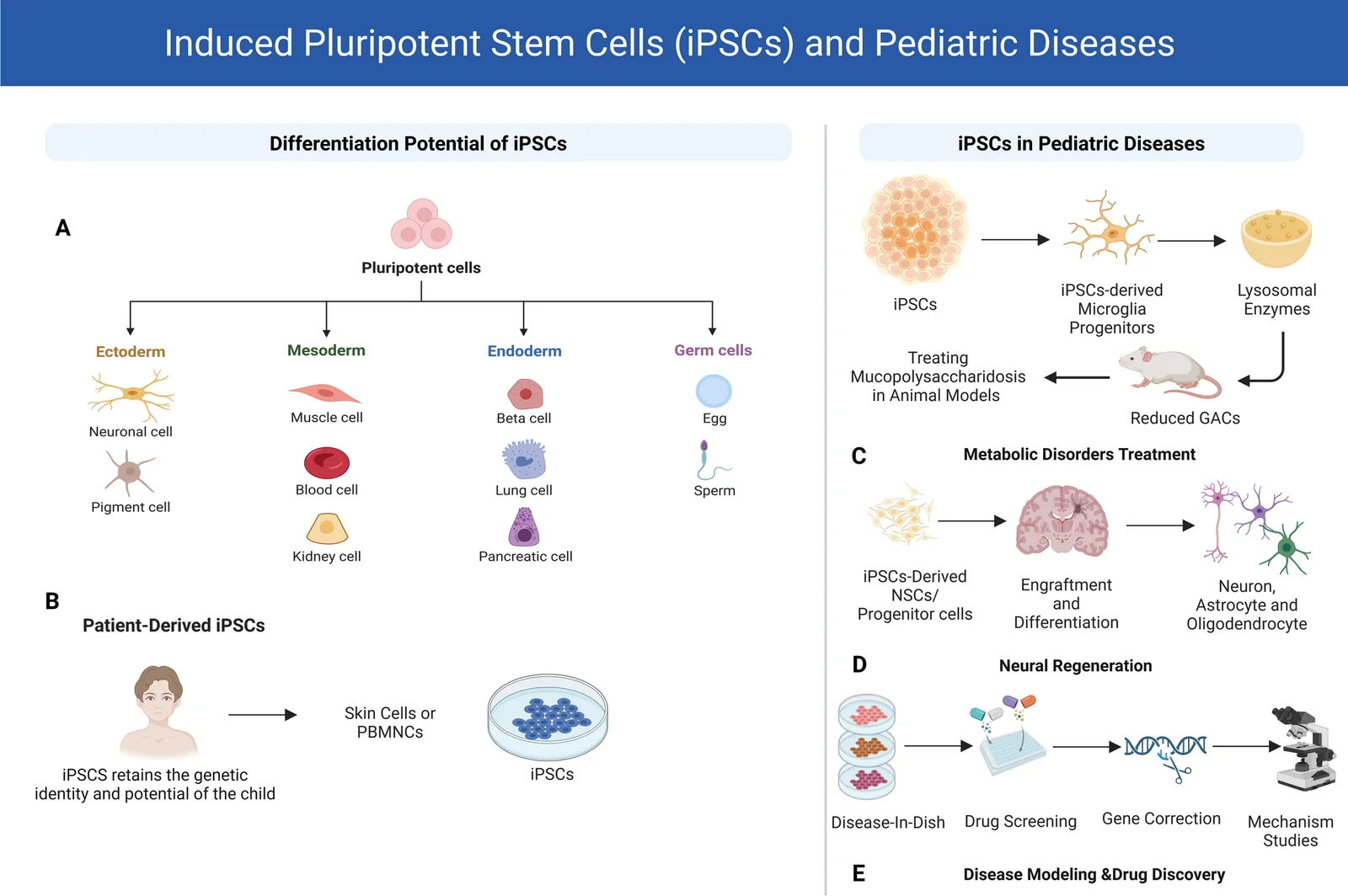
Figure. Induced pluripotent stem cells (iPSCs) and their applications in pediatric diseases. **A** iPSC’s capabilities allow them to convert into cells of any of the three germ layers in addition to germ cells, demonstrating their vast potential in cellular development because of their pluripotent nature. **B** iPSCs are generated from somatic cells obtained from patients (e.g. fibroblasts and PBMNCs obtained from children). Because iPSCs are derived from patient cells, they maintain a child’s genetic identity, enabling the creation of an animal model of the disease and the testing of therapeutics in the child. **C** iPSC-derived microglial progenitors and derivatives of other lineages will be used to examine lysosomal enzyme deficiencies (e.g. mucopolysaccharidoses) and use them to assess therapeutics in nonhuman primate models. These studies have led to a decrease in the accumulation of GAGs. **D** The use of iPSC-derived NSCs provides ways to engraft NSCs and then differentiate into neurons, astrocytes, and oligodendrocytes, thus allowing the repair of damage done to the nervous system as part of a potential treatment plan for children with a neurological disorder. **E** iPSCs can provide a “disease-in-a-dish” platform to develop targeted therapies to treat an entire class of diseases through the use of high-throughput drug screening and/or testing for gene corrections and understanding the cellular processes that lead to disease. *This conceptual framework was adapted from previously published methodologies and synthesized from insights from key foundational and translational studies that collectively cover iPSC generation*,* disease modeling*,* and pediatric applications *[106–112]

Using patient-derived cells has reduced many of the ethical concerns associated with using embryos as a source of cell production. Cells harvested from (autologous) patients who generate their own (genetic) material will also lessen any chances of that patient rejecting those cells due to tissue typing issues [25, 26]. Improvements in differentiation protocols enable faster, reproducible production of immune cells derived from iPSCs, including T cells and macrophages. For example, newly developed next-generation iPSC platforms will improve access to cell-based immunotherapy for pediatric cancer patients and children with primary immunodeficiencies; however, clinical translation of these technologies remains evolving [27]. There are numerous issues with iPSCs that must be resolved before their use in therapy. These include problems with genome stability during reprogramming, the potential for oncogene activation during reprogramming, and the significant difficulties in expanding iPSCs for human therapeutic use to levels acceptable for clinical manufacturing standards. Before human patients (especially children, who continue to grow up until their mid-20s) can be treated with iPSCs, they must undergo rigorous preclinical safety assessments (Fig. 2) [26].

### Mesenchymal stem cells: clinical translation and safety

Stem cell populations that have been extensively studied in pediatric research and trials include mesenchymal stromal cells (MSCs). Multipotent cells are found in bone marrow, umbilical cord, placenta, and adipose tissue and have been extensively studied because of their immunomodulatory properties, low immunogenicity, and good safety record [28, 29].

Over the last 5 years, numerous clinical trials have evaluated MSC therapy in children across a variety of indications, including respiratory, cardiac, orthopedic, neurologic, endocrine, and hematologic conditions. Specifically in pediatric neurology, MSCs have been evaluated in conditions such as CP (cerebral palsy), ASD (autism spectrum disorder), and stroke, where it has been hypothesized that MSCs may act through anti-inflammatory mechanisms, support endogenous repair, and produce trophic & immunomodulatory substances [30, 31].

The safety record of MSC therapy in children is supported by published research. Current evidence supporting MSC therapies is predominantly focused on demonstrating safety and feasibility than on proving that they help produce positive clinical outcomes. Although researchers have reported evidence of A MSC therapy having clinically beneficial effects in some studies, the findings have not produced consistent results across various disease indications and patient populations. The application of MSC therapy to treat immunological conditions has not been a singular point of concern. Phase I/II trials evaluating the safety and effectiveness of umbilical cord-derived MSCs in children with multidrug-resistant nephrotic syndrome have shown safety, and the therapeutic efficacy of this approach is currently being determined [28].

Extracellular vesicles derived from mesenchymal stromal cells are being studied as a new approach to treat newborns with various illnesses. Hwang and colleagues examined MSC-derived extracellular vesicles to determine whether they possess anti-inflammatory and regenerative properties in severe neonatal disease and, therefore, have potential as a substitute for administering whole-cell MSCs [29].

Pediatric respiratory disorders have been studied for the use of mesenchymal stromal cells as a treatment, due to their ability to improve lung damage associated with lung injury and influence immune responses. Similar to this study, preclinical studies using iPSC models were also conducted on adult patients to further investigate how these cell types can be used to evaluate regenerative and immunomodulatory therapies [32]. The clinical outcomes of mesenchymal stromal cell use have not always been favorable. In a Phase IV study, umbilical-cord-derived MSCs administered with traditional immunosuppressive therapy were found to be safe but did not demonstrate short— or long-term clinical benefit in children newly diagnosed with severe aplastic anaemia [28].

Regenerative medicine is afflicted by a continuing issue of having good safety but no demonstrable benefit. The contrast of preclinical findings being positive and clinical outcomes demonstrating no direct benefit requires the need for greater numbers of randomized studies, more standardized potency assays, and enhanced methods of stratification of patients. The following new paragraph has been added: This study also highlights an important principle in regenerative medicine: A favorable safety profile does not guarantee a measurable clinical benefit. The disparity between the favorable results in preclinical studies and the null result in clinical trials underscores the need for larger randomized clinical trials, standardized potency measures, and improved patient stratification before widespread use can be justified. Significantly, the research demonstrates the common problem that regenerating medicine is often not without risk. However, evidence of continued safety profiles will not guarantee positive clinical outcomes. Furthermore, the difference between seemingly promising data from preclinical research and non-significant data from clinical trials suggests that large randomized clinical trials need to be conducted using standardized potency tests, appropriate patient stratification and adequate rigor to examine the long-term efficacy of these interventions prior to routine clinical use.

**Fig. 3:**
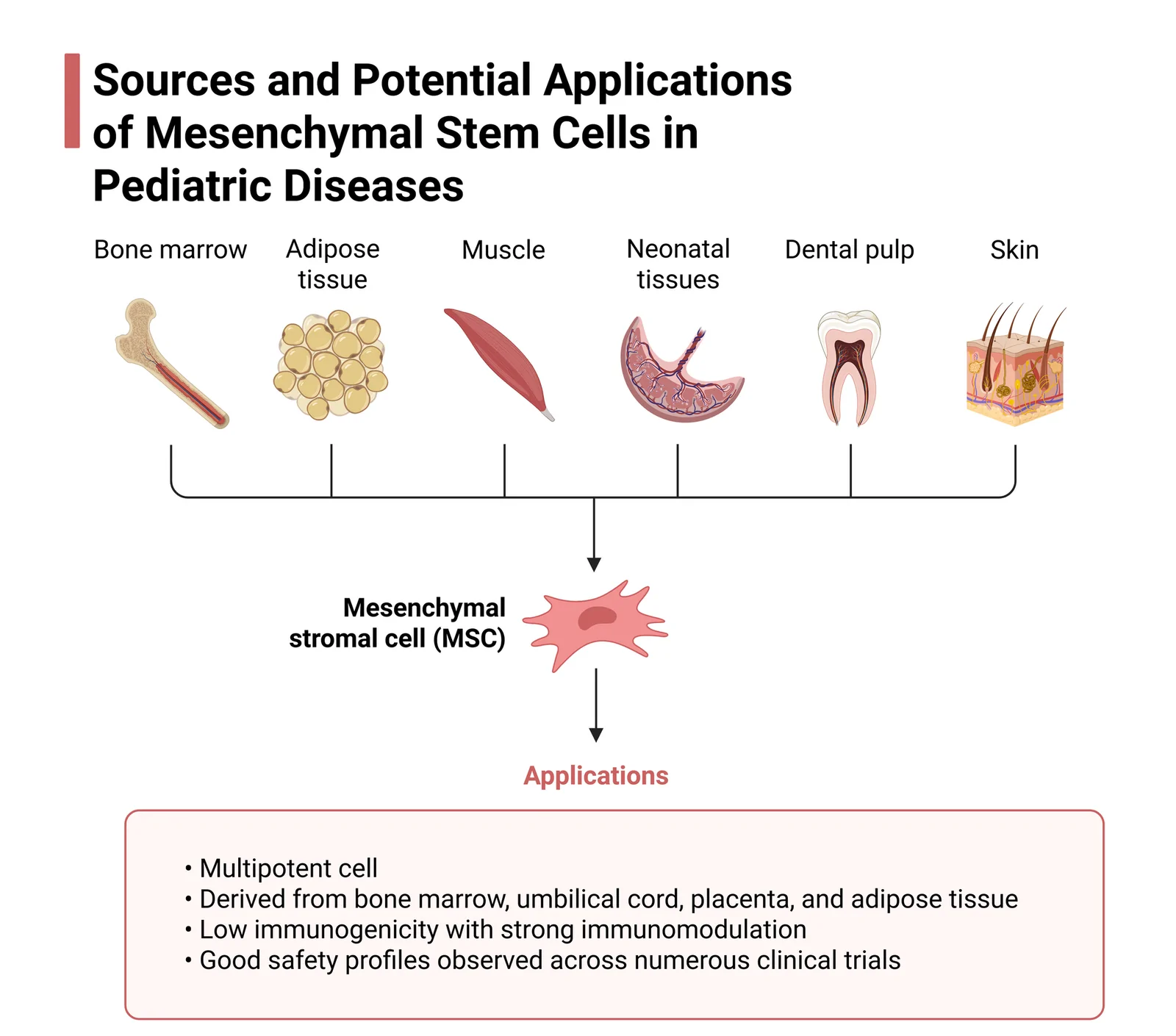
Possible Sources and Uses of MSCs for a Range of Conditions in Children. Bone marrow, adipose tissue, skeletal muscle, tissue from new babies (umbilical cords and placentas), dental pulp, and dermis. Regardless of their origin, MSCs are multipotent and can modulate the immune system. MSCs have minimal immunogenicity, strong paracrine, and immunomodulatory effects; therefore, they are an attractive target for therapeutic intervention in pediatric diseases. Clinical and preclinical studies provide strong safety data across a variety of diseases, making them a viable treatment option for regenerative medicine and conditions arising from altered immune responses in children. *This conceptual framework was adapted from previously published methodologies and synthesized from insights from foundational and translational work describing MSC sources*,* biological properties*,* and clinical use—particularly in immunomodulation and pediatric contexts *[105, 107, 113, 114] *The schematic design used in* Fig. 3* was created using a BioRender template similar to that used in a previous publication *[115]

In pediatric research, MSCs are one of the most heavily studied stem cell sources today. Despite many studies demonstrating that MSCs generally have a good safety profile, their clinical efficacy varies widely depending on the disease being treated. Multiple factors across studies, including donor source, manufacturing methods, dose administration routes, and patient outcome assessment methods, lead to substantial variation in reported results for MSC-based therapies. Hence, while MSCs are considered among the most clinically advanced platforms for regenerative medicine currently being studied, there have been difficulties in reaching firm conclusions regarding the effectiveness of MSCs in a range of pediatric disorders. However, efficacy and standardization require continued study to assess their suitability for clinical use (Fig. 3). Hematopoietic Stem Cells (HSCs) are one of the most studied types of stem cells for use in pediatric medicine. Many studies have shown that HSCs have a good safety profile; however, there is significant variability in their clinical effectiveness based on the specific disease type. Differences in the donor source, the method used to manufacture HSCs, dosing, route of administration and the way in which outcomes are measured all contribute to the large degree of variability seen in the clinical trial results.

### Hematopoietic stem cells (HSCs): the foundation of stem cell therapy for children

**Fig. 4:**
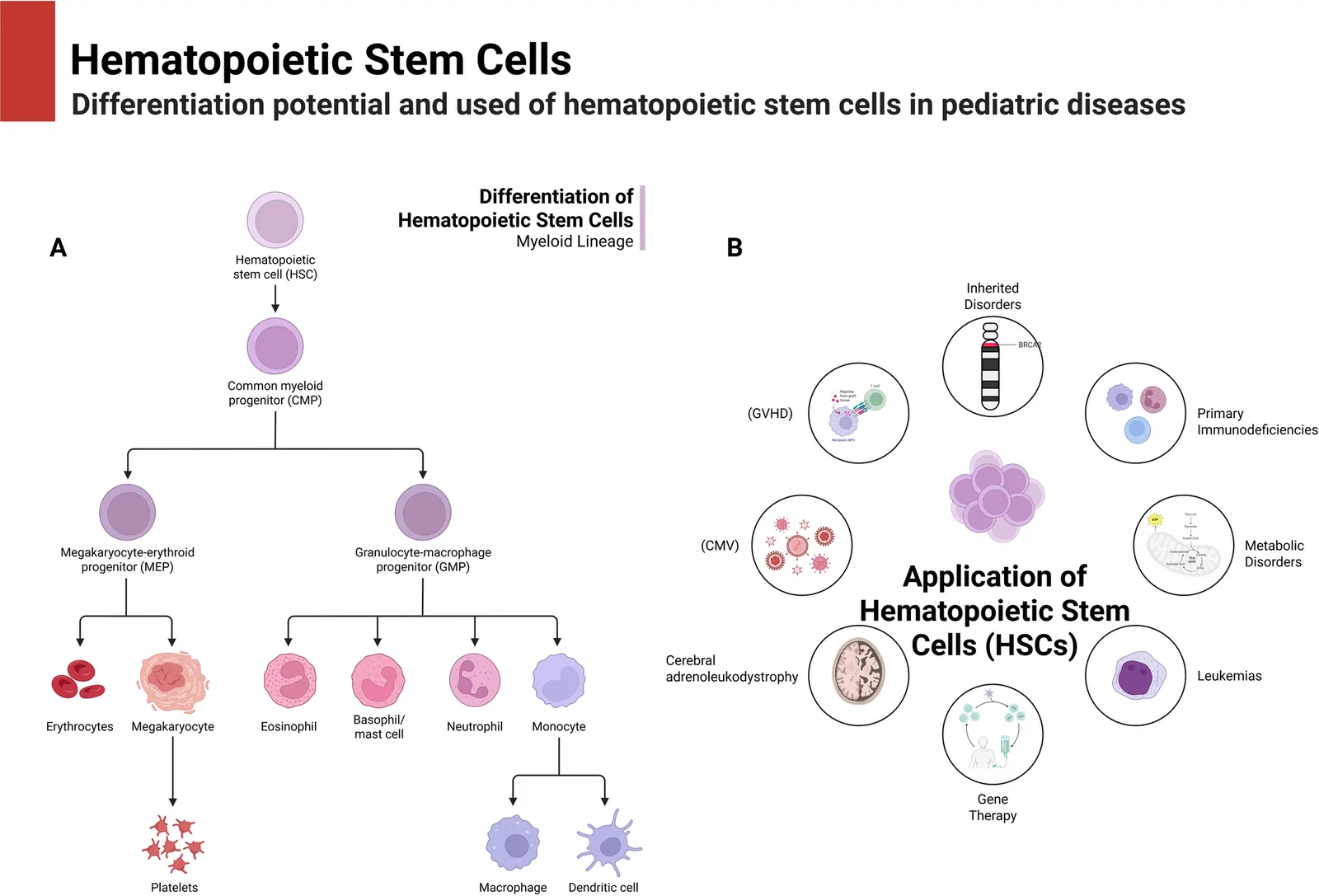
Differentiation potential and clinical applications of hematopoietic stem cells (HSCs) in pediatric diseases. HSC(s) are very similar to mature/specialized cells emitted from the self-renewing & differentiating HSC(GMP).HSC are used as an experimental platform to develop novel therapies for pediatric patients, where they can be administered in an allow setting to provide HSCs or other cell therapy approaches for the treatment of a wide range of hematological disorders (primary immunodeficiency disorders, genetic & metabolic conditions, leukemias, cerebral adrenoleukodystrophy); also using HSC as an in vivo vehicle for gene therapy efforts targeting monogenetic disease processes. Clinical considerations include potential complications such as graft-vs-host disease (GVHD) and opportunistic infections (e.g., Cytomegalovirus). *This conceptual framework draws upon established methodologies and integrates insights from foundational and translational research related to hematopoietic stem cell biology*,* lineage differentiation*,* transplantation*,* and gene therapy (McCulloch and Till in the 1960s; Weissman*,* 2000; Appelbaum*,* 2007; Daley*,* 2015; Kohn*,* 2016; EBMT guidelines; NIH resources) *[105, 107, 113, 114]

Hematopoietic stem cells (HSCs) are the basis of stem cell therapy in pediatric practice and can be collected from bone marrow, peripheral blood, and umbilical cord blood. HSCs are routinely used to treat inherited hematological disorders, primary immunodeficiencies, leukemias, and selected metabolic diseases in children. Allogeneic hematopoietic cell transplant (HCT) is a well-established therapeutic option for neonates and infants with leukemia. Clinical studies, including Kostareva et al., have demonstrated the effectiveness and potential clinical benefit of allogeneic HSC transplantation in this high-risk patient population [33]. Cytomegalovirus (CMV) infections and graft-versus-host disease (GVHD) remain major clinical challenges in pediatric patients undergoing allogeneic hematopoietic stem cell transplants (HCT). Despite the significant risk of developing either CMV infection or GVHD (or both) after transplantation, HCT remains an important life-saving therapy for many patients at high risk of dying from their illness, regardless of whether they are otherwise healthy or have multiple other serious co-morbidities. To achieve optimal outcomes, it is critical that transplant-related complications be managed comprehensively; therefore, transplant centers should implement strategies to prevent GVHD and treat its complications effectively [34]. Because of their ready supply and ability to regenerate, umbilical-cord-derived hematopoietic stem cells (HSC) are becoming increasingly popular as an alternative source for transplants. Umbilical-cord blood (UCB) has been associated with a lower frequency and/or lower severity of graft-versus-host disease (GVHD) and greater tolerance to major histocompatibility complex (MHC) mismatches than other hematopoietic stem cell sources, making it a particularly good choice for pediatric transplantation [35]. An example is elivaldogene autotemcel for disease progression to slow in boys with cerebral adrenoleukodystrophy by using gene therapy with autologous HSCs. This shows how genetically modified HSCs may be therapeutically beneficial for treating a pediatric genetic disorder [35]. Hematopoietic Stem Cells (HSCs) are a quintessential, translatable platform in the field of pediatric medicine and have led to the development of many novel FDA approved & investigational therapies (Fig. 4).

### Tissue-specific progenitor cells (Neural, Cardiac, Hepatic)

Tissue-specific progenitor cells exhibit limited differentiation capacity and have been examined as candidates for regenerative medicine, alongside pluripotent and multipotent stem cells. They are often regarded as a viable option for reducing the risk of inappropriate differentiation and cancer formation, owing to their commitment to a particular lineage. Neural stem and progenitor cells constitute a lineage currently under investigation for congenital and acquired central nervous system diseases and disorders, including hypoxic-ischemic injury, spinal cord injury, and certain neurodegenerative diseases, thereby providing opportunities for pediatric translational research [36–40]. Initial clinical observations indicate that mesenchymal stem cells derived from fetal tissues may assist patients with motor skills, communication, and electroencephalography on a case-by-case basis. Studies are now being conducted to determine how safe and effective the implantation of the two types of stem cell (hematopoietic and mesenchymal) in a child’s spine and spinal fluid will be in treating cerebral palsy [41]. Cell-based therapies have been evaluated in pediatric cardiology for mechanisms of action that employ cardiac progenitor cells and other heart-derived stem cell sources to treat pediatric patients. The purpose of this systematic review/general meta-analysis was to analyze the safety and/or therapeutic effects of preclinical and clinical studies (predominantly involving mesenchymal stromal cells) of cell-based therapies for children with congenital or acquired heart diseases. The majority of studies appear to demonstrate some degree of improvement in cardiac function, as evidenced by increases in ejection fraction; however, additional studies are needed to validate these findings [42]. The use of stem cell-derived hepatocyte-like cells for transplant may be an interesting new research field in response to inherited metabolic disorders of the liver. These methods have not yet reached the clinical level, but they can provide an alternative to blood (hematopoietic) and fat (mesenchymal) based stem cell therapies by using induced pluripotent stem cells or other pluripotent sources. If successful, they would represent a future option for the treatment of children with liver disorders who would otherwise need a liver transplant [28]. In addition to working on cardiac and neurological cells, pediatric research has examined tissue-specific progenitor cells derived from the lungs, pancreas, kidney, and skeletal muscle. A recent review suggests that modified umbilical cord-derived mesenchymal stromal cells may affect disease progression in pediatric lung diseases; however, clinical validation remains ongoing [32, 42].

In conclusion, stem cell therapy in pediatric medicine presents both opportunities and challenges. Ethical and regulatory concerns limit the use of embryonic stem cells despite their ability to differentiate into a wide variety of cell types. Induced pluripotent stem cells address ethical concerns associated with embryo use and enable patient-specific therapies. However, ongoing challenges remain in safety, genomic stability, and the ability to manufacture these cells at large scale. Currently, mesenchymal stromal cells are the most versatile and have the best safety profiles for use in pediatrics, whereas hematopoietic stem cells remain the primary means of treating patients with hematological and genetic diseases. Progenitor cells from specific tissues, such as the brain, heart, and liver, are increasingly being used in regenerative medicine for pediatric patients.

The long-term use of each cell type will depend on the type of disease, the patient’s age, risk tolerance, and the results of long-term follow-up studies. To realize the full therapeutic potential of stem cells in children, extensive preclinical testing, well-designed clinical trials, and appropriate regulatory and ethical oversight must be established. It is critical to recognize that stem cell platforms can differ from one another based on their current amounts of clinical evidence. While hematopoietic stem cell transplants create a clear standard for treatment of many pediatric hematologic and immune disorders, mesenchymal stem cell therapy is still being evaluated for use in treating patients. In addition, induced pluripotent stem cells, extracellular vesicles, and organoid technology (the type of technology used to produce organs) up to this point have primarily been studied at the preclinical or early translational levels; therefore, the degree of acceptance of any therapeutic claim must fit within the level of clinical validation, regulatory maturity, and longer-term safety record for the specific stem cell product (Table 1).

**Table 1:** Hierarchy of evidence and clinical maturity of stem cell platforms in pediatric medicine

Stem cell platform	Representative pediatric applications	Current clinical status	Strength of evidence	Major limitations	References
Hematopoietic Stem Cells (HSCs)	Leukemia, primary immunodeficiency, inherited metabolic disorders, sickle cell disease	Standard clinical practice	High	GVHD, infection risk, donor availability, conditioning toxicity	[33, 129, 130]
Gene-Modified HSCs	Cerebral adrenoleukodystrophy, hemoglobinopathies, inherited immunodeficiencies	Regulatory approval or advanced clinical development	High–Moderate	Manufacturing complexity, cost, long-term surveillance	[31–133]
Mesenchymal Stromal Cells (MSCs)	GVHD, cerebral palsy, inflammatory disorders, bronchopulmonary dysplasia	Advanced clinical investigation	Moderate	Variable efficacy, product heterogeneity, lack of standardization	[49, 134, 135]
Umbilical Cord Blood Products	Neurological injury, immune disorders, transplantation support	Clinical investigation	Moderate	Limited cell dose, inconsistent efficacy data	[73, 97, 136]
Tissue-Specific Progenitor Cells	Neurological, cardiac, hepatic disorders	Early clinical investigation	Low–Moderate	Limited long-term data, manufacturing challenges	[30, 44, 137]
Induced Pluripotent Stem Cell (iPSC)-Derived Therapies	Monogenic diseases, disease modeling, regenerative medicine	Predominantly preclinical or early translational	Low	Tumorigenicity, genomic instability, scalability	[26, 27]
Extracellular Vesicles (EVs)	Neonatal injury, inflammatory disorders, neurological repair	Early-phase clinical investigation	Low	Lack of potency assays, dosing uncertainty, manufacturing variability	[75, 138, 139]
Organoids and Bioengineered Constructs	Disease modeling, personalized medicine, regenerative scaffolds	Experimental	Very Low	Regulatory uncertainty, limited clinical validation	[61, 98]

One important consideration is that the stem cell platforms do not have the same level of clinical validation at this farther along than others. Hematopoietic stem cell transplantation, for example, will provide an established standard of care for many children with hematologic, immunologic, and metabolic diseases and has decades of clinical data and follow-up evidence supporting its use. However, there are currently many application types of cell therapies with respect to the mesenchymal stromal cell therapies; therefore, they are actively involved in clinical trials, whereas the extracellular vesicles, induced pluripotent stem cell-derived products, organoid systems, and many bioengineered stems cell therapies will still be considered preclinical or early in their transitional cycle. Because of these variations, the interpretation of therapeutic claims should also take into consideration the degree of clinical validation of each platform, its regulatory maturity, and the amount of long-term safety data available (Table 1).

Although tissue progenitor cell therapies have provided positive results in preclinical and early translational studies, the existing studies to date are limited by small sample sizes, insufficient clinical experience, and lack of large-scale validation studies. As a result, any conclusions made about their efficacy and long term safety must be made with caution. Validation studies performed independently, multicenter studies, and adequately powered clinical trials will be needed to provide sufficient evidence for the reproducibility, long term safety and clinical usefulness of these therapies prior to their broad implementation [43–45].

## Mechanistic insights into pediatric stem cell therapy

**Fig. 5:**
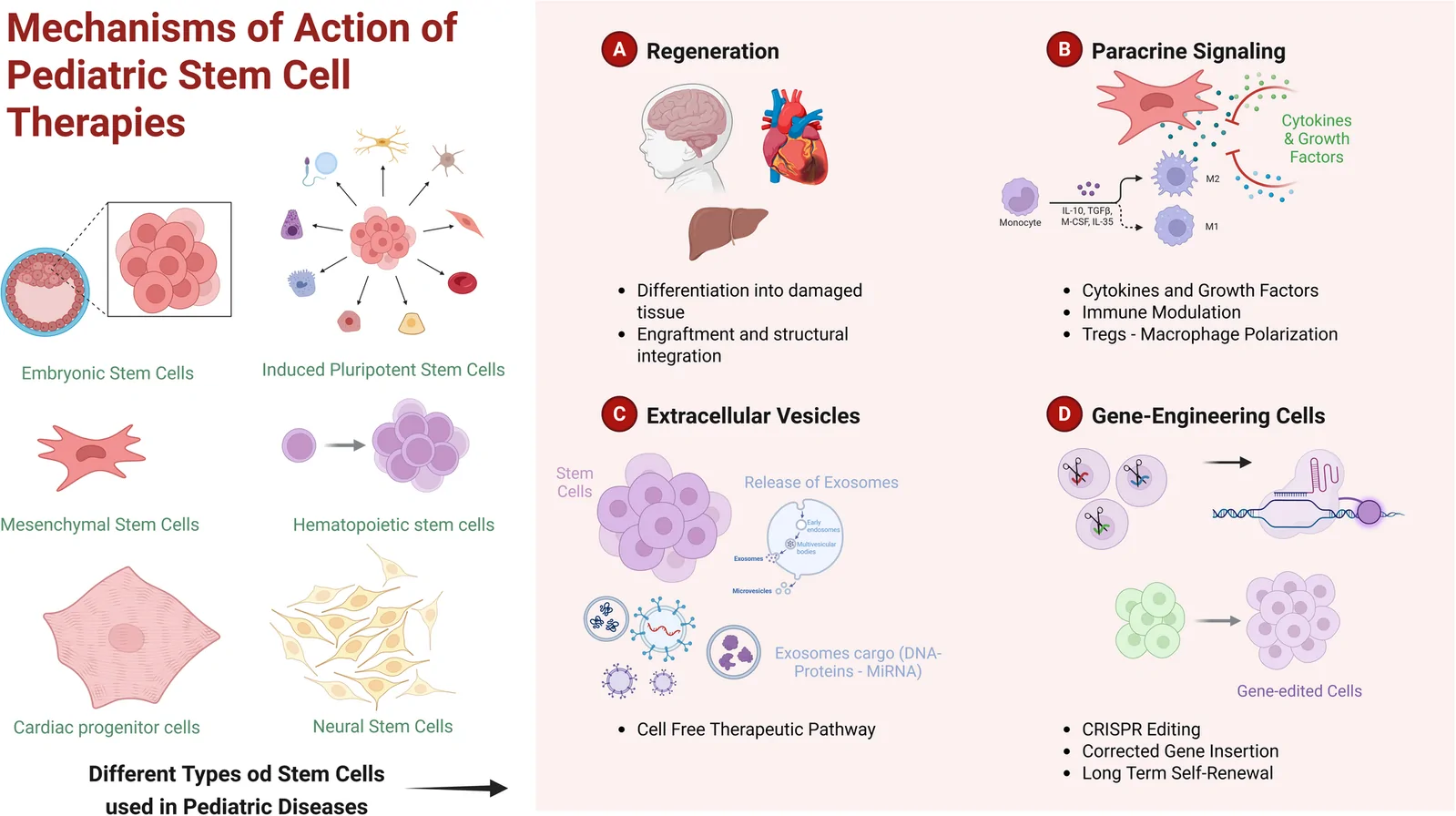
Main Mechanisms of Stem Cell Therapy in Pediatric Patients. Stem cells can provide therapeutic effects through four primary methods of action: (1) direct differentiation and integration into tissues for regeneration; (2) through paracrine signaling via the secretion of cytokines, chemokines and growth factors; (3) through the use of extracellular vesicles to allow intercellular communication, delivering bioactive cargo; (4) through durable biological repair through gene correction or bioengineering of cells. All of the above mechanisms are context-dependent based on both the stage of development, type of inflammatory responses present, and type of diseases present. *This conceptual framework is adapted from other methodologies previously published and is synthesized from foundational and translational studies describing stem cell mechanisms of action (regenerative differentiation*,* paracrine signaling*,* extracellular vesicle biology and gene-engineered therapies *[25, 104, 106, 112, 116, 117]

Stem cells are also used in pediatrics for non-cell-replacement mechanisms. To develop an optimal therapy, we need to better understand these mechanisms in relation to maximizing patient safety and long-term benefit. In the continuing sections, we will describe the various mechanisms by which current stem cell therapies may work. These mechanisms fall into four main categories: tissue regeneration, paracrine signaling, extracellular vesicle signaling, and gene modification/engineering of stem cells prior to patient administration. Stem cell treatments seldom use a single approach but often employ multiple mechanisms of action in an integrated manner (e.g., repairing tissue, modulating the immune system, and correcting molecular defects). This creates a multidimensional treatment profile for pediatric patients, who typically have more complex physiologies than adults (Fig. 5).

### Regenerative and reparative potential

The intrinsic regenerative capacity of stem/progenitor cells is a key factor in stem cell therapy for infants/children. MSCs are multipotent mesenchymal stromal cells that promote tissue regeneration in distinct ways depending on the mechanism of their initial injury. This means that these cells can migrate to sites of injury, remain in an inflammatory environment, and promote tissue repair by either differentiating into other cell types or stimulating resident cells to proliferate [28]. Stem cells can be used as a source of repair for pediatric illnesses characterized by tissue loss or organ dysfunction. Stem cells have been used in hematopoietic cell transplantation (HCT) to support tissue repair and immune system balance by increasing engraftment success rates while decreasing the incidence of graft-versus-host disease using mesenchymal stromal cells (MSCs) [46]. In metabolic or neurodegenerative pediatric disease models, stem or progenitor cells can restore structure and function, often by integrating into existing host tissues or acting through trophic mechanisms.

Importantly, the regenerative process is context-dependent: stem cells modulate their behavior in response to microenvironmental cues, dynamically shifting between differentiation, survival, and the secretion of reparative signals. Homogeneity of stem cell migration and engraftment may be influenced by the interaction of the stromal-derived factor-1/CXCR4 signaling pathway. Through the signaling pathway between CXCL12 and CXCR4, hematopoietic stem cell trafficking to bone marrow niches occurs, as well as the maintenance of engrafted stem cells in damaged tissues. The expression levels of CXCL12 and CXCR4 can vary greatly depending on tissue type, developmental stage, and disease state; therefore, the variability of these factors may explain some of the variability of success after administration of stem cell therapies [47, 48].

### Paracrine mechanisms and immunomodulation

One of the most critical insights from the last decade is that paracrine signaling, rather than direct engraftment or differentiation, is frequently the dominant mechanism by which MSCs mediate therapeutic benefit [30, 49].

MSCs secrete a rich secretome—cytokines, chemokines, growth factors, microRNAs, even mitochondria—that act on nearby cells to reduce inflammation, promote survival, and modulate immune responses [50] This paracrine crosstalk allows MSCs to suppress overactive immune reactions, such as in GVHD, and to encourage a healing environment. In pediatric settings, where modulating immune responses is often as critical as regenerating tissue, this mechanism is particularly valuable. Molecular pathways that are used by MSC for immunomodulation are multiple. The TGF-beta promotes the differentiation of regulatory T cells while also helping to suppress the activation of the immune response so not too much happens. The IDO inhibits T cell proliferation by removing tryptophan from the environment. In contrast, PGE2 helps the macrophage become polarized towards an anti-inflammatory phenotype. Other mediators, such as IL-10 and HGF, also act in combination with IDO and PGE2 to inhibit an inflammatory response and promote tissue repair. As such, these molecular pathways likely work together in a coordinated and context-dependent manner and would not have a single dominant pathway by which to accomplish their goals [51, 52].

Furthermore, MSCs exhibit immunomodulatory plasticity, tailoring their response to the inflammatory milieu [32]. For instance, they can shift immune cells toward anti-inflammatory phenotypes (e.g., inducing regulatory T cells or polarizing macrophages toward a pro-resolving state), a behavior highly relevant when treating pediatric inflammatory or autoimmune disorders [53]. While we have learned a lot about MSC biology, we do not yet understand how important each mechanism is. Many of these proposed mechanisms have been found in laboratory settings but have not been consistently observed in human studies with children. Therefore, mechanistic explanations should be used carefully when trying to apply results from preclinical studies to the clinic.

### Extracellular vesicle–mediated signaling and therapeutic relevance

Soluble factors and extracellular vehicles (EVs), which include exosomes and microvesicles, are key mediators of the activity of mesenchymal stromal cells. EVs are membrane-bound vesicles that carry bioactive materials, such as proteins, lipids, messenger RNA, and micro-RNA, to recipient cells. EVs also enable targeted communication between cells within recipient tissues [31].

The seminal work of Hwang et al. on MSC-derived EVs supports their therapeutic utility in preclinical neonatal and pediatric models. This approach has anti-inflammatory, anti-apoptotic, and reparative properties while minimizing the potential risks of live-cell transplantation, such as immunogenicity and tumor formation [54]. There is now a lot of information indicating that micro-RNAs that are carried by an extracellular vesicle may play a major role in how that vesicle behaves biologically. The most commonly cited examples are miR-21, miR-146a, and miR-124, which mediate inflammatory signaling pathways, cell death pathways (apoptosis), macrophage polarization pathways, and pathways involved with neural repair. While these three examples are the most often studied, individual micro-RNA contributions remain poorly defined since there is tremendous variability among the micro-RNA compositions of EVs (extracellular vesicles) prepared from different cells and produced by different methods [55, 56].

In preclinical brain injury models, the neurologic outcome improved, seizure activity decreased, and white matter integrity was maintained following systemic administration of extracellular vesicles produced by MSCs. This provides evidence that cell-free vesicle therapy may mimic the important therapeutic benefits of conventional cellular MSC therapies.Moreover, EVs may enhance safety and scalability in pediatric populations: they can be produced, stored, and dosed more like biologic drugs than living cells, thereby reducing the complexity of administration and associated risks. Theoretical benefits exist for EV-based therapy; however, these therapies are still at an early stage of being developed clinically. There are numerous barriers to development - such as lack of standardized methods of EV isolation, potency assays, manufactural homology, dosing parameters, & long-term safety studies. Therefore, EVs will currently be viewed as experimental products yet to reach the level of clinical proven therapies at this time [29]. One unanswered question is whether the total therapeutic activity of extracellular vesicles (EVs) can be matched by their parent cells. Compared to traditional cell-based therapies, EVs may be more secure, and also be produced at a much larger scale than these types of intervention—however, there is not enough comparative data available from clinical studies to determine how best to create EVs (in terms of manufacturing and dosing) to correlate as closely as possible with the parent cells’ therapeutic activity.

In spite of the fact that extracellular vesicles have the potential to become an attractive therapy in a cellular-free manner, there is limited current evidence available from the clinic. As nearly all pediatric-based applications are still under investigation in early phases, conclusions regarding their effectiveness are preliminary until they have been validated through larger, controlled studies.

### Gene-corrected and bioengineered stem cells

Pediatric treatment is undergoing evolution with the use of gene-corrected and bioengineered stem cells, which provide a combination of sustained engraftment and targeted molecular repair of disease-causing genetic changes. Hematopoietic stem cells, for example, have been genetically modified to correct genetic defects that cause inherited diseases and to generate long-term, self-renewing cell populations that can restore normal function to the blood and immune systems by expressing corrected genes [35] .This approach provides a one-time, curative potential, crucial in pediatric settings where lifelong therapy may otherwise be required.

The ability to generate patient-specific, autologous iPSCs provides an excellent therapeutic option. The modification of the genome using established gene editing techniques like CRISPR for correcting the pathogenic variant in the patient’s cells is accomplished with precision and then can be directed into the desired lineage (e.g., neuronal or hepatic) and returned to the patient. This promise of a new type of medicine is fraught with regulatory and safety issues, but the potential to cure numerous monogenic pediatric diseases without the risk of immunological rejection is substantial [22, 57]. Successful differentiation and integration of neural cells is often dependent on developmental signalling pathways like the Wnt/β-catenin, Notch and Sonic Hedgehog (SHH) pathways. These signalling pathways play a role in the regulation of stem cell self-renewal, lineage commitment, neural patterning, and maturation of tissues. The manipulation of these signalling pathways has improved the efficiency of differentiation in research, however, inappropriate activation of these pathways could also lead to an increased risk of abnormal differentiation or tumour formation [43, 58].

Utilization of bioengineering methods may provide unique advantages for pediatric regenerative medicine. Pediatric patients have enhanced endogenous regenerative ability compared to adults and often have a stronger ability to repair damaged tissues, which may improve the effectiveness of regenerative therapies.[42] Pediatric patients also typically have less cumulative tissue injury and fibrosis compared to adults and develop in highly dynamic developmental microenvironments that may improve the efficacy of engineered cellular products and regenerative interventions [59, 60]. These biological features may contribute to improved tissue integration, remodeling, and functional recovery after receiving regenerative therapies and bioengineered tissue constructs [61]. However, the interaction of engineered cellular products with developing tissues is still poorly defined, and thus warrants additional studies through pediatric-specific translational studies.

Another option would be to place stem cells within biomaterial scaffolds to improve stem cell viability, promote directed migration, and enable controlled release of growth factors. Therefore, this bioengineering technique is based on underlying biological principles to improve the quality and duration of therapy [62, 63].

Gene-edited stem cells, and stem cells modified using bioengineering, are exciting advancements toward effectively using stem cells for their regenerative capabilities. We still face a number of challenges related to gene-edited stem cells such as (i) off-target genomic effects; (ii) incomplete gene editing; (iii) complexity of producing stem cells; (iv) regulatory oversight of manufactured stem cells; and (v) undefined long-term safety of using stem cells in the pediatric population.

In conclusion, umbilical stem cell therapy acts through multiple mechanisms beyond direct cell replacement, including tissue regeneration, paracrine signaling, extracellular vesicle-mediated communication, and engineering-based modification. By identifying the core domains, better, safer, more precise, and appropriate therapies for children can be developed using a rational approach.

In order for bioengineered stem cell therapies to be effectively used in children, it is necessary that these therapies are shown to be effective, but that more than this is done when addressing a child’s developmental biology or growth outcomes and the long-term safety of the product after treatment.

### Developmental considerations unique to pediatric stem cell therapy

Children are not simply smaller adults. Developmental stage significantly influences stem cell homing, engraftment efficiency, immune recognition, tissue remodeling, and long-term therapeutic outcomes. The pediatric immune system undergoes continuous maturation throughout infancy, childhood, and adolescence, creating age-dependent differences in immune tolerance, inflammatory responses, and graft persistence. These developmental factors may influence both therapeutic efficacy and safety and should therefore be considered when interpreting pediatric stem cell studies [42, 64].

Furthermore, endogenous regenerative capacity differs substantially between children and adults. Developing tissues often possess greater regenerative potential and more plastic microenvironments, which may enhance responsiveness to regenerative therapies. However, these same developmental characteristics may also increase susceptibility to unintended effects, including aberrant differentiation, altered tissue growth, or disruption of normal developmental pathways. The interaction between transplanted cells and the evolving pediatric tissue niche remains incompletely understood and represents an important area for future investigation [28, 46].

Dosing strategies and routes of administration also require pediatric-specific consideration. Optimal cell dose may not scale directly according to body weight, as developmental physiology, tissue size, vascular distribution, and disease-specific factors may influence biodistribution and therapeutic activity. Similarly, the safety and efficacy of intravenous, intrathecal, intramuscular, and local tissue delivery approaches may differ substantially across age groups and disease states. Despite increasing numbers of pediatric stem cell trials, standardized age-specific dosing recommendations remain largely unavailable [59].

Long-term monitoring is particularly important in pediatric populations because cellular therapies may persist for years or decades following treatment. Surveillance strategies should therefore include assessment of growth, neurodevelopment, immune function, tissue remodeling, and potential late adverse effects, including ectopic tissue formation and tumorigenicity. Similar concerns have been raised for emerging extracellular vesicle-based therapies, where long-term pediatric safety data remain limited despite encouraging early clinical findings [29].

These considerations highlight the importance of developing age-specific clinical trial designs, outcome measures, dosing strategies, and long-term follow-up protocols tailored to pediatric populations. Future progress in pediatric regenerative medicine will depend not only on improving therapeutic efficacy but also on understanding how developmental biology influences stem cell behavior throughout childhood and adolescence [28, 46].

## Clinical applications and translational advances

### Induced pluripotent stem cells (iPSCs)

Induced pluripotent stem cells (iPSCs) are revolutionizing how scientists study pediatric diseases because they allow for the development of patient specific tissue from an individual’s own cells derived from the skin (or another source) while still maintaining the genetic characteristics and developmental history of the child, giving the iPSC equal potential as the child for use in regenerative approaches to pediatric neurodevelopmental and neurodegenerative disorders. Recent studies have provided substantial evidence that human iPSC-derived neural stem/progenitor cells can stably engraft in the brains of host animals and differentiate into glial lineages without evidence of tumor formation, making them a strong basis for developing regenerative treatments for pediatric neurodevelopmental and neurodegenerative disorders [65]. Another study demonstrates how iPSC-derived microglia progenitors can deliver deficient lysosomal enzymes to multiple severe mucopolysaccharidosis animal models of disease and minimize excessive accumulation of the pathological form of glycosaminoglycans associated with these diseases. This process may open new avenues for developing therapeutic products derived from iPSCs to treat these critical metabolic disorders that arise early in childhood [66]. In addition to providing therapeutic options for developing and conducting future research on disorders, iPSCs can be used as “disease-in-a-dish” models, enabling researchers to study and understand the underlying mechanisms of disease in children; develop new therapeutics; screen for new drugs; and develop gene-correction strategies in vitro. The combination of these findings provides strong evidence of the broad and varied potential of iPSC technology to improve understanding of pediatric disease and to lead to transformative therapies (Fig. 2). The possibility of iPSC-derived therapeutics is well established based on prior results of both disease modelling as well as proof-of-concept studies; however, very few iPSC-derived therapeutics have undergone advanced stage clinical evaluation. Consequently, preclinical success shouldn’t be misconstrued to indicate upcoming timeliness of their use in clinical settings.

### Hematological and immune-system disorders

The clinical management of pediatric hematologic and immunologic disorders has changed to include stem cell-based treatment approaches. Mesenchymal Stromal Cells (MSCs) have been studied for their immunomodulatory effects in patients undergoing allogeneic Hematopoietic Stem Cell Transplantation (HSCT) when being treated for graft-versus-host disease (GVHD). The results from early-phase and observational studies demonstrate an acceptable safety profile and evidence of clinical response over time when MSCs are administered alongside other therapies (non-MSCs) for ASTM-GVHD. Long-term follow-up data from some patients indicated improved survival and symptom outcomes [49]. These findings are supported by concurrent pilot studies of decidual stromal cells in cases of acute GVHD—cells that appear to exhibit a vibrant immunosuppressive profile [46]. Success observed in HSCT for myriad metabolic leukodystrophies, including metachromatic leukodystrophy, with supplementary MSC administration suggests that stromal cells modulate not only the alloimmune toxicity process but also the neuroinflammatory milieu, thereby exacerbating disease symptoms [67]. Recent studies evaluating human umbilical cord-derived mesenchymal stromal cells and cord blood-derived hematopoietic progenitor cells have been reviewed as adjuncts or alternatives for treating severe aplastic anemia and congenital immunodeficiency disorders. Preliminary findings indicate they could have favorable proliferative capability and low immunogenicity; this may support engraftment and minimize the risk of transplant-related morbidity/increased mortality; however, very few controlled clinical trials exist to substantiate these claims [35, 68]. With numerous CAR T-cell therapies undergoing phase II and III clinical trials, there have been many success stories within the pediatric population who have experienced remission after being treated for either relapsed or refractory acute lymphoblastic leukemia. In addition, ongoing initiatives are focused on developing additional stem-cell-derived and genetically-modified immune effector therapies to improve the durability, safety, and reproducibility of these treatment strategies [27, 69]. These studies, taken together, point to a critical theme: stem cell-based treatments contribute not only to the hematopoietic transplant process but also modulate the immunologic context, enabling chronic remission of fatal childhood hematologic diseases (Fig. 3).

### Neurological and neurodevelopmental disorders

**Fig. 6:**
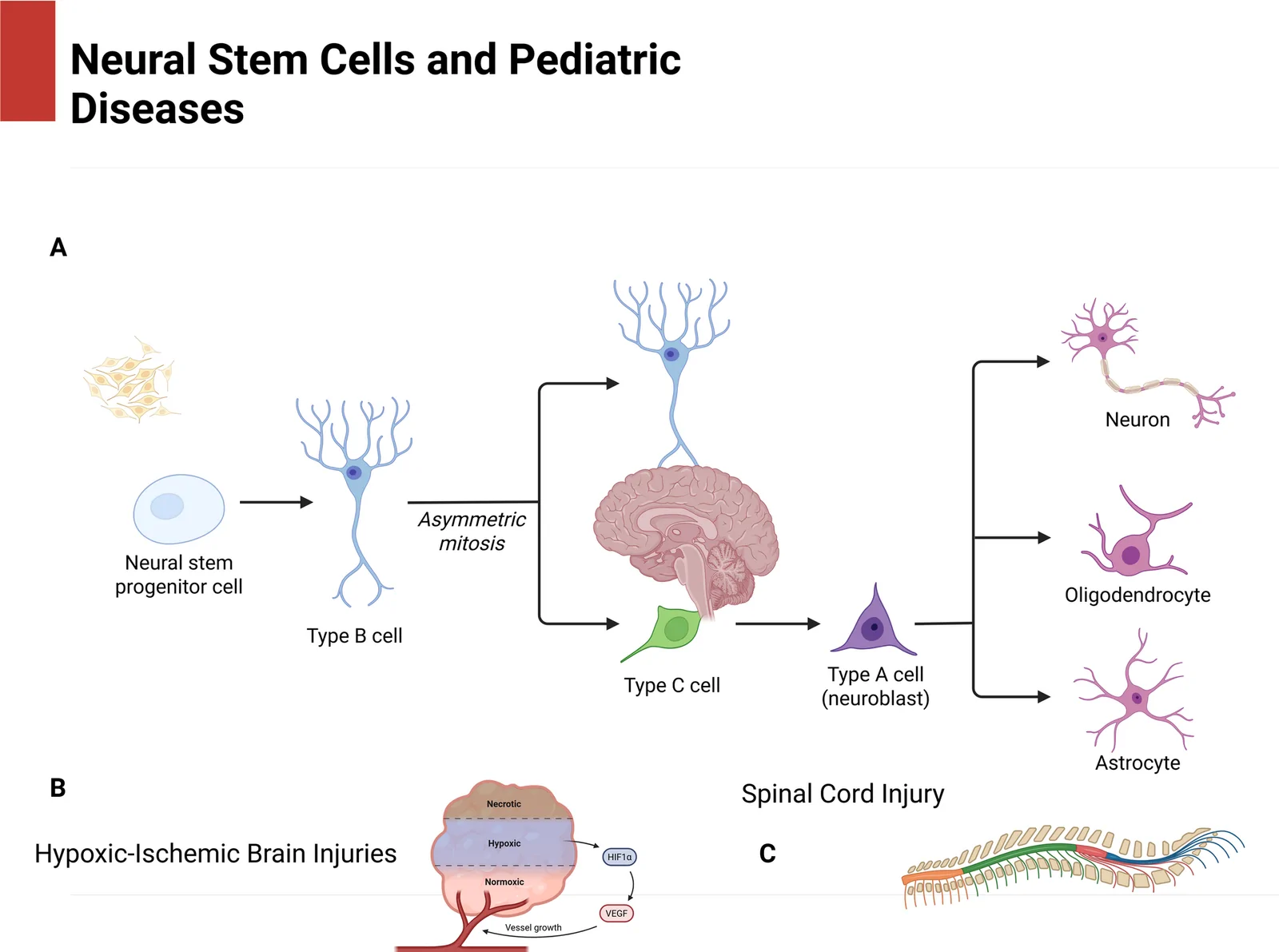
Neural stem cells and their relevance in pediatric neurological disorders. **A** Progression of lineage of neural stem/progenitor cells. Neural stem/progenitor cells will give rise to type B cells, which divide asymmetrically to keep the stem cell pool while also producing intermediate progenitor cells (type C). Type C cells will ultimately differentiate into type A cells (neuroblasts), which mature into the three major neural lineages: neurons, oligodendrocytes, and astrocytes. The hierarchical organization ensures that neurodevelopment (neurogenesis) and gliodevelopment (gliogenesis) will continue throughout both the developing and postnatal brain. **B** Possible role of NSC in the brain injury process caused by hypoxia and ischemia. Injury to the brain due to oxygen deprivation leads to the formation of distinct regions of necrosis and hypoxia in the tissue of the brain that is injured. Activation of hypoxia-inducible factor (HIF-1α) will activate vascular endothelial growth factor (VEGF), which will promote new vascularization (angiogenesis) and remodel the injured tissue. In addition, NSC strategies to enhance repair may include NN replacement by cell replacement, trophic support, and/or modulation of the microenvironment of the injured tissue. **C** Use of NSCs as a possible therapy for people with spinal cord injury. Transplanted neural stem or progenitor cells may promote recovery of both structure and function by differentiating into neurons or glia and by enhancing axon regeneration, remyelinating axons, and re-establishing neural connections. These strategies have the potential to improve outcomes for those who suffer from spinal cord injury and related neurodevelopmental disorders in children. *The conceptual framework that was developed in this research study is based upon a number of prior studies published by others related to neural stem cell biology and its role in the development of new neurons; lineages of specialized cells; how hypoxic conditions affect structural integrity in the central nervous system; and how these systems produce new neurons to assist with recovery from neurological diseases *[118–121]

Numerous clinical trials are under way investigating stem cell interventions for children with neurological disorders, and since children have a greater capacity for recovery from injury due to brain development, there may be benefits to using these types of therapies. Mesenchymal stromal cell therapies have been studied in preclinical models as well as in early-stage trials, and there is a growing body of evidence that shows they can modulate injury from excess excitation, reduce activation of microglia, and maintain connections between neurons [70]. There has been progress on clinical translation of pediatric cerebral palsy, including numerous completed phase I/II and randomized trials. Recent systematic reviews have indicated potential enhancement of motor functions for some children treated with therapies using mesenchymal stromal cells; however, the ability to draw firm conclusions from these findings is hindered by significant variability in study design, type of cell source, method of manufacture, treatment protocol, method of measurement, duration of follow up, and bias risk. Though evidence does provide some promising outcomes, a reliable rationale for effectiveness has yet to be claimed [31]. Subjective measures of improvement are reported in case reports using umbilical-derived MSCs, with functional improvements ranging from increased tone regulation to cognitive engagement [71]. Perinatal brain injury continues to be a key area for stem cell-based treatment targeting a major cause of lifelong disability. Recent trials of umbilical cord blood-derived mononuclear cells in neonatal hypoxic-ischemic encephalopathy have shown clinical improvements, including better electrophysiological recovery and developmental scores [72]. Initial studies involved direct utilization of allogenic cord-derived MSCs to newborns with hypoxic injury, further supporting the safety and feasibility of this approach [73]. In all of these conditions, the mechanisms converge: immunomodulation, trophic support, metabolic stabilization, and extracellular vesicle-mediated tissue repair together shape the context for a brain repair response (Fig. 6) [51, 74–76]. Importantly, there is a lack of always documented consistent benefit from therapy with studies due to variances in treatment response, outcome measure used, as well as the way the research trials have been performed, and ultimately this hampers our ability to interpret the current evidence and ultimately limits the ability for independent confirmation of the efficacy of the therapy being assessed.

The inadequacy of most studies to frequently show therapeutically beneficial responses. Disparate treatment responses; lack of basic clinical trial standardization; differences in clinical trial methodology; varying outcome measure; the variable duration of follow-up make it difficult to interpret current clinical trial literature. The variability of treatment response shows that further research is warranted. Therefore, additional properly designed and adequately powered, double blind, controlled clinical trials must be completed prior to assessing efficacy.

### Cardiovascular and metabolic pediatric diseases

**Fig. 7:**
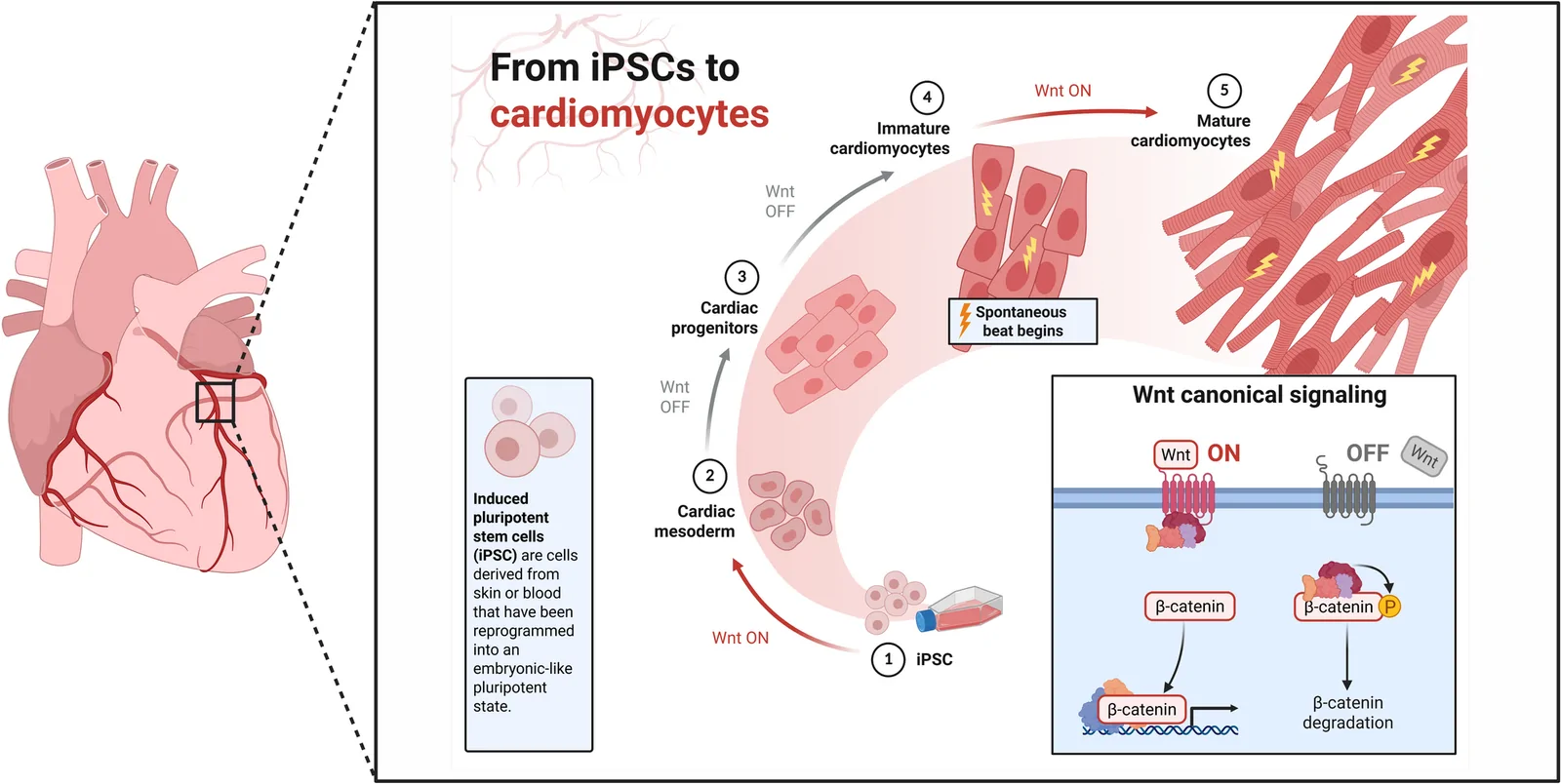
Directed differentiation of induced pluripotent stem cells (iPSCs) into cardiomyocytes through modulation of Wnt signaling. Diagrammatic representation depicting the progress of generating cardiomyocytes (CMs) from human induced pluripotent stem cells (iPSCs). Induced pluripotent stem cells (1) from somatic cell sources, reprogrammed to iPSCs, are temporally treated with canonical Wnt signaling to produce CMs. Activation of canonical Wnt signaling (Wnt ON) from exposure to Wnt ligand(s) induces the transition to cardiac mesoderm (2), after which time Wnt is inhibited (Wnt OFF), resulting in the differentiation of cardiac mesoderm cells into cardiac progenitor cells (3). After further maturation, immature CMs develop (4) with the initiation of spontaneous contractile activity, followed there after by the ultimate development of mature CMs (5) that possess organized sarcomeric structures, and have enhanced electrophysiological characteristics. To differentiate CMs from iPSCs efficiently, the temporal regulation of the Wnt/β-catenin signaling pathway is critical. During the Wnt ON state, Wnt ligands bind to their receptors and thus stabilize β-catenin and promote its translocation into the nucleus, where β-catenin activates gene transcription of target genes. When Wnt is turned OFF, β-catenin is phosphorylated and targeted for ubiquitin-mediated proteasomal degradation, thereby preventing downstream signaling. *This conceptual framework was adapted from previously published methodologies and synthesized from foundational studies on pluripotent stem cell differentiation and stage-specific modulation of Wnt/β-catenin signaling during cardiomyocyte development *[25, 122–126] *The schematic design used in* Fig. 7*was generated using BioRender templates similar to those employed in the following publications *[127, 128]

In children with congenital heart disease, cardiac repair is complicated by the presence of structural defects, the child’s metabolic vulnerability and continuing myocardial growth. The scientific literature and clinical studies on progenitor-progenitor based therapy were reviewed to evaluate adjunctive mesenchymal stromal cell therapies. The systematic reviews demonstrate modest improvement (increase in ejection fraction) of ventricular function with the use of MSCs or other related MSC products [77]. The potential benefits of therapy for patients with severely impaired ventricular function, ranging from those with single-ventricle anatomy to patients with post-surgical cardiomyopathy who respond poorly to standard treatment, appear to be more evident in children than in adults. Although the use of stem cells as a therapeutic option for the treatment of inherited metabolic disorders due to enzyme deficiencies is still in its infancy, stem cell-based approaches have been considered in specific conditions, including the use of hematopoietic stem cell transplantation with additional cellular supplementation as an adjunctive therapy for metachromatic leukodystrophy, which is being assessed in early-stage clinical trials [67]. Recent clinical studies have led researchers to hypothesize that combining cellular therapies will be able to complement the reconstitution of deficient metabolic pathways and maintain neurological function. Similarly, investigational strategies using induced pluripotent stem cells (iPSCs) to generate immune or metabolic lineages will enable the creation of patient-specific, genetically modified cellular products that address the underlying molecular deficiency; however, such strategies remain largely preclinical or at early stages of development [27]. Collectively, these studies suggest emerging perspectives on stem cell therapy, as reparative biology and metabolic engineering have the potential to rescue organ systems previously beyond the reach of other stabilization avenues after irreversible decline (Fig. 7).

### Musculoskeletal, dermatological, and rare genetic disorders

Researchers are investigating stem cell-based treatments for chronic injuries involving inflamed tissues that do not heal properly in juvenile patients with rheumatologic and musculoskeletal diseases. Mesenchymal stem cells (MSCs) have been tried as a therapy for the treatment of severe cases of juvenile arthritis. Studies conducted at a single site or at a limited number of sites provide evidence that this treatment has an anti-inflammatory effect and can improve physical functioning; however, larger studies with more participants are needed to substantiate these findings [53]. While not supported by extensive amount of high-quality evidence, the possibility to use MSCS to modulate inflammation, fibrosis, angiogenesis, and repair of skin and connective tissue diseases is also under consideration. Some early clinical reports have noted the application of MSCs to treat neonates with rare genetic diseases associated with abnormalities of structural proteins or disruption of normal inflammatory pathways. In addition, umbilical cord-derived stromal and hematopoietic products are still being studied in early-phase trials for a range of musculoskeletal conditions, including cerebral palsy [31, 71]. Though these results indicate possible therapeutic action, the current state of knowledge is still deficient because most studies have small sample sizes of patients, used varying study designs (due to poor standardization) and have demonstrated differing clinical efficacy levels. Thus, further randomized, controlled studies with sufficient power must be performed in order to determine efficacy. While some early clinical trials indicate there may be a benefit, there is not enough evidence to prove that the treatment is effective for a wide range of musculoskeletal disorders in children, and it is also possible that the published study results reflect only successful outcomes.

### Overview of ongoing and completed clinical trials

Judging by all currently posted pediatric stem cell trials, we continue to see an increase in number, as well as a diverse range of research being conducted in this area. A review of the major global registries has provided evidence to confirm ongoing studies with mesenchymal stromal cells (MSCs), hematopoietic stem cells (HSCs), umbilical-derived products and engineered iPSC-based products across multiple indications/conditions such as nervous system disorders, blood/immune system disorders, heart/lung (cardiopulmonary), kidney (renal) or metabolic disorders [64]. Most registered pediatric stem cell trial studies are in the early phases, as found in the analysis of the registered pediatric stem cell trials. Further, many have focused on safety and feasibility instead of efficacy outcomes. Trial methodology and long-term follow-up framework disparities among disease populations, cell sources, study designs, and outcome measures exist; therefore, standardized methodologies for each study are needed [64]. Commentary states that the number of trials has increased among newborns and infants, targeting diseases such as neonatal encephalopathy, congenital cardiac disease, bronchopulmonary dysplasia, and immunologic diseases [32, 72, 78]. The majority of ongoing pediatric clinical trials in stem cell therapy remain authorized at phases I or II to evaluate safety and feasibility, whereas studies in later phases are beginning to examine the functional and developmental outcomes of these therapies, which are important for clinical practice. Efforts to develop new treatments continue, with some studies shifting from whole-cell therapeutics toward the use of both extracellular vesicle-based and genetically modified cellular therapies that are currently undergoing phase I or II studies [27, 29, 75, 76]. and AI-guided cell optimization, from initial considerations to cellular wound mechanics [79] are increasingly being used in clinical studies, including stem cell studies.

Clinical trials show there is much still to be learned in terms of pediatric stem cell research, as the majority of current published studies are still being conducted relative to safety and feasibility, with less emphasis on assessing overall efficacy. In pediatric stem cell trials, researchers need to analyze positive and negative studies as well as neutral findings when drawing conclusions about the success of stem cell therapy. The presence of strong biological rationale for the use of stem cells but lack of consistent clinical efficacy is one of the biggest challenges being faced by researchers in the field of translational regenerative medicine (Table 2).

**Table 2:** Representative completed and ongoing clinical trials evaluating stem cell-based therapies in pediatric disorders

Disease/condition	Cell platform	Trial phase	Primary endpoint	Major findings	References
Steroid-Refractory GVHD	MSCs	Phase II	Safety and GVHD response	Favorable safety profile; variable efficacy	[135, 140, 141]
Cerebral Palsy	MSCs/Cord Blood Cells	Phase I–III	Motor function improvement	Modest functional improvements reported; heterogeneity remains substantial	[31, 142, 143]
Neonatal Hypoxic-Ischemic Encephalopathy	Umbilical Cord-Derived Cells	Phase I–II	Safety and neurodevelopment	Feasible and generally safe; efficacy under investigation	[144–146]
Severe Aplastic Anemia	Umbilical Cord MSCs	Phase IV	Hematologic recovery	Safe but failed to demonstrate significant clinical benefit	[99, 147]
Infant Leukemia	HSC Transplantation	Standard of Care	Overall survival	Established therapeutic benefit	[33, 148]
Cerebral Adrenoleukodystrophy	Gene-Modified Autologous HSCs	Approved Therapy	Disease stabilization	Significant slowing of disease progression	[149, 150]
Metachromatic Leukodystrophy	HSC-Based Gene Therapy	Approved/Advanced Clinical Use	Neurological preservation	Improved outcomes when administered early	[131, 151]
Pediatric Immunodeficiency Disorders	Gene-Corrected HSCs	Early Clinical Trials	Immune reconstitution	Promising preliminary outcomes	[131, 152, 153]
Bronchopulmonary Dysplasia	MSCs	Phase I–II	Safety and pulmonary outcomes	Early evidence of safety; efficacy remains investigational	[32, 154]
Neonatal Disorders	MSC-Derived EVs	Phase I	Safety	Feasible; efficacy not yet established	[75, 78, 139]

Data summarized from published trials and registry analyses

## Challenges in pediatric stem cell therapy

Pediatric use of stem cell-based therapies presents unique challenges. Children have biological differences from adults as children are at different levels of development, have different immune responses, and have different susceptibility to long-term adverse effects. Therefore, children will need to be evaluated based on their age. New advances in cell sourcing, delivery, and manufactured standards must continue to progress as we develop clinical implementation of stem cell therapies; however, regulatory, safety and ethical complexities will need to be addressed in a thorough, systematic manner so that they can be used by all patients in a safe and effective manner.

### Ethical and regulatory issues specific to children

Pediatric stem cell therapy has an extremely regulated ethical framework due to children’s inability to give informed consent as well as due to the fact that many of the children being treated have diseases for which there is no accepted medical treatment. Therefore, the regulatory form should be based on a risk vs. benefit analysis with respect to the treatment’s clinical efficacy/potential; the analyses are especially critical for products that do not have a current medical indication or clinical efficacy, as is the case for pluripotent and genetically modified products. While there are reports of potential therapeutic activity results from studies of interventions using mesenchymal stromal cells to treat inflammatory and neurological disorders, there remains a continued emphasis on the need to protect this vulnerable population by maintaining strict ethical and regulatory oversight [28, 53, 70]. Many view umbilical cord blood as very safe and ethically acceptable for the donor. Its increased popularity is partially due to its extraction from a donor population that is not harmed [35]. Nevertheless, some ambiguity exists regarding donor anonymity, ongoing biobanking responsibilities, and the potential use of cells for secondary platforms beyond their originally intended purpose. New products enter the market, including formulations derived from extracellular vesicles [29, 76]. Regulatory assessments need to evolve to ensure product safety while appropriately categorizing products without compromising innovation.

### Immunological compatibility and graft longevity

Immunological concerns remain the primary reason pediatric patients are treated with stem cells. The immune system is continually developing and can exhibit diverse response patterns, from highly reactive to highly tolerant, making predictions of possible outcomes very difficult. During allogeneic HSCT, pediatric patients remain at risk for developing graft-versus-host disease (GVHD), even with additional mesenchymal stromal cell (MSC) infusions [27, 46, 49]. Clinicians often assert that MSCs are “immune evasive.” Still, recent work suggests that their immunomodulatory effects may not be permanent, and repeated administration does not guarantee sustained clinical outcomes [68]. Using umbilical-derived MSCs and cord blood mononuclear cells, often thought to have more lenient HLA restrictions, minimizes, though does not eliminate, practical issues [72]. The addition of genetically engineered or iPSC-derived immune cells introduces further complications regarding immunogenicity and the potential for delayed rejection [27]. Pediatric patients, with their developmental and extended life spans ahead, require grafts that can be tolerated in the near term and that also engraft, persist, and function appropriately within a developing immune system over the subsequent childhood and adolescent years.

### Tumorigenic potential and prolonged follow-up

The tumorigenic risk is an essential factor to consider in pediatric stem cell research. Mesenchymal stromal cells have shown a relatively good safety profile in trials evaluating cerebral palsy, metabolic disorders, and brain trauma; however, concerns remain about the risk of malignant transformation, ectopic tissue growth (growth in an abnormal location), and dysregulated growth. Furthermore, these risks are likely to be greater with pluripotent or gene-edited stem cell products because residual undifferentiated cells may retain pathogenic potential [27]. There are only limited long-term outcome data regarding pediatric stem cell treatments. Ongoing monitoring is essential to evaluate delayed adverse effects after receiving either an autologous or an allogeneic (non-related) stem cell product. Some patients with refractory T-cell Graft Versus Host Disease and those with leukodystrophies have been followed for prolonged periods. This has provided valuable information; however, it is still insufficient to fully understand the long-term safety profile of cellular therapy [49, 67]. However, these examples imply and expose the extent of the commitment required from both families and health systems as new cell classes are incorporated into the clinical setting, such as formulations based on extracellular vesicles [52, 78]. We have to remain vigilant and assess when the boundaries of tumorigenic risk may need to be re-evaluated.

### Manufacturing, reproducibility, and quality control

Manufacturing safe, cellular-based products for children is technically challenging. The variability among donors, the source of tissue used, and the production facility can all affect the potency, purity, and viability of the product. Furthermore, with the strict regulations surrounding Good Manufacturing Practice (GMP), products like mesenchymal stromal cells (MSCs) are complex, dynamic, self-renewing cell populations that respond to inflammatory, hypoxic, and mechanical stimuli and may, therefore, introduce variability in their therapeutic effects [51, 74]. Work continues to establish manufacturing pipelines; the efficiency of adding surveillance-quality assurance cannot be achieved quickly enough. Advances in AI and systems-based modeling provide new frameworks for managing differentiation pathways, tracking batch-specific characteristics, and identifying potency measures prior to transplantation [80]. Nonetheless, it remains challenging for international centers to produce comparable products, particularly for complex ones such as immunogenic cells derived from iPS cells or neonatal-evolved EV formulations [27, 78]. The need for stringent manufacturing and quality control is substantially increased in pediatric patients, whose dose thresholds and safety margins differ markedly from those of adults.

In addition to scientific and regulatory challenges, cultural, social, and economic factors also influence the successful implementation of pediatric stem cell therapies. Acceptance of stem cell-based therapies by the public may differ between regions based on cultural and ethical values, religious beliefs about stem cells, the perception of new biomedical technologies, and attitudes toward specific types of stem cells (especially embryonic stem cells) [81, 82]. Moreover, there are significant disparities in healthcare systems, regulatory frameworks, and available financial resources, all of which will affect the availability of advanced regenerative therapies [83, 84].

Pediatric medicine presents unique challenges in terms of ethical consideration when determining how to balance parental rights and the welfare of the child being treated. As such, ethical considerations must account for the long-term safety of the child and equity in access to healthcare [81, 82]. The high costs associated with the production, quality assurance, and long-term follow-up associated with advanced cellular therapies may also contribute to the existing gaps in access to care [83, 84]. Future translational efforts should incorporate the tenets of health equity, affordability, and global access in order to ensure that the advancements in regenerative medicine will benefit all pediatric populations, not just those who live in countries with ample healthcare resources [85, 86]. Collaboration among researchers, clinicians, policymakers, regulatory agencies, advocates, and other global healthcare stakeholders will be necessary to create ethical, sustainable, and equitable frameworks for the clinical application of pediatric stem cell therapies [86].

### Barriers to clinical trial design and recruitment into pediatric trials

Pediatric clinical trial design and conduct both involve ethical protection requirements and limited numbers of potential subjects. Recruitment for studies may also be challenging for rare or ultra-rare conditions due to the limited number of available subjects and their geographic dispersion. Consequently, the studies may end up underpowered, requiring long enrollment periods and/or using either adaptive or multicenter designs to conduct relevant analyses [64]. Families may be discouraged from participating in early-phase research studies for many reasons, including safety issues, logistical challenges associated with the study, and emotional strain on the family members who would have to agree to participate. Also, the lack of standardization for endpoints remains an issue. Because neurologic, cardiometabolic, and musculoskeletal disorders often lack immediate or easily quantifiable outcomes, determining whether treatment is effective is difficult. The variability in the design of studies, selection of endpoints, and methodology for measuring outcomes in studies of children with heart disease and children with cerebral palsy provides examples of this variability [31, 77]. The development of standardized, age-appropriate endpoints that facilitate meaningful comparisons across studies to help validate products is becoming more important to regulatory agencies. While ongoing methodological and other barriers limit the number of pediatric stem cell studies, the number of such studies conducted globally has increased significantly. Registry data showing that cell-based therapies are being evaluated in clinical trials has increased as well, including research looking into therapies for neonatal encephalopathy, congenital metabolic conditions, immune dysregulation, and refractory inflammatory conditions [64]. However, ongoing issues of recruitment, long-term follow-up, and standardized protocols continue to create barriers to translation. The multitude of challenges should not be considered separate hindrances to accomplish transition to pediatric stem cell therapy, but rather, as interdependent forces driving successful clinical translation. While each of these complexities, such as scientific uncertainty, production variability, regulatory standards, ethical considerations, and trials’ design limitations separately influence the ability to achieve them successfully, they also collectively affect how feasible, scalable and ultimately sustainable the pediatric stem cell treatment can be. To overcome these obstacles will necessitate a collaborative effort between physicians, researchers/scientists, industry stakeholders, regulatory authorities and groups representing patients. Table 3 summarizes the major obstacles to widespread application of stem cell therapies for children, along with strategies that might be used to overcome them.

**Table 3:** Major translational barriers and proposed solutions in pediatric stem cell therapy

Challenge	Clinical impact	Proposed solutions	References
Tumorigenicity and Ectopic Differentiation	Long-term safety concerns	Enhanced purification methods, genomic surveillance, prolonged follow-up	[42, 70]
Immune Rejection and Graft Failure	Reduced efficacy and durability	Improved matching strategies, immune engineering, tolerance induction	[46, 155, 156]
Manufacturing Variability	Poor reproducibility between studies	Standardized GMP protocols, potency assays, centralized production systems	[139, 155, 157]
Lack of Standardized Potency Assays	Difficulty comparing products	International consensus guidelines and validation studies	[88, 139, 158]
Limited Long-Term Pediatric Data	Unknown delayed adverse effects	National and international patient registries	[42, 131, 159, 160]
Small Trial Sizes	Reduced statistical power	Multicenter collaborations and adaptive trial designs	[28, 31, 77]
Ethical Issues and Consent	Challenges in pediatric enrollment	Enhanced parental counseling and ethical oversight	[35, 161]
Socioeconomic Inequities	Limited access to therapy	Global partnerships and equitable reimbursement strategies	[35, 79, 90]
Regulatory Complexity	Delayed clinical implementation	Harmonization of FDA, EMA, and international regulatory pathways	[35, 79, 162]
High Cost of Advanced Therapies	Restricted accessibility	Manufacturing optimization and value-based reimbursement models	[97, 162, 163]
Cell Delivery Challenges	Inadequate tissue targeting	Biomaterials, engineered scaffolds, and targeted delivery technologies	[61, 98, 139]
Lack of Pediatric-Specific Endpoints	Difficulty assessing efficacy	Development of age-specific outcome measures and registries	[77, 134, 160]

### Regulatory translation and clinical implementation

More than biological effectiveness is needed for successful translation of therapeutics utilizing stem cells in children. The future translation of pediatric stem cell therapies will rely on the different scientific validation, standardized manufacture, regulatory oversight, ethical compliance, and sustainable healthcare delivery systems together as each of these different components have been identified as critical to the successful translation of cell therapy [52, 87]. Advanced pediatric stem cell therapies will require robust quality control, reproducibility in manufacturing, and harmonized regulatory pathways to ensure product safety, consistency, and scalability [88]. Ethically, the informed consent process, long-term follow-up & monitoring, equitable access to therapies, and protection of vulnerable pediatric populations must all be included in a translational plan rather than be developed in isolation from the scientific development [42]. The future success of these therapies will depend on the establishment of a coordinated and comprehensive framework for translational activities so that all of these related issues can be addressed simultaneously and not as separate problems [89].

There have been a number of major developments in the area of stem cell research; there are numerous interrelated obstacles preventing this area from translating into routine clinical practice within the field of pediatrics. To make continued progress in the field of stem cell therapy for children, future successes will require technological advancements and the development of standardized manufacturing, defined clinical trial methodologies, long-term safety monitoring, equitable access frameworks, and harmonization of regulatory processes. Each of these elements’ forms part of the roadmap for developing the next generation of pediatric regenerative therapies.

The issues considered in the course of this review (i.e., safety monitoring, standardization of manufacturing processes, regulatory oversight and ethical governance) feature various aspects which are interrelated; these issues require addressing through coordinated/translational frameworks, not by means of isolated interventions.

The successful translation of stem cell therapies into clinical practice is contingent upon compliance with increasingly complex regulatory frameworks aimed at ensuring the safety, consistency and efficacy of stem cell-based products [83, 90]. Regulatory agencies such as the U.S. Food and Drug Administration (FDA) and the European Medicines Agency (EMA) currently utilize risk-based evaluation of Advanced Therapy Medicinal Products (ATMPs) for granting approval and include Good Manufacturing Practices (GMP), product characterization, potency testing, batch-to-batch consistency and long-term follow-up of patients as part of their respective regulations [83, 91].

Because the majority of stem cell-based products are biologically complex with a natural variability, regulatory approval will increasingly be based not only upon demonstrating clinical benefit but also on demonstrating reproducibility and the quality assurance of the manufacturing process [92, 93].

To assist the practical translation of stem cell-based therapies, a roadmap for standardization of manufacturing processes, validated potency assays, comprehensive preclinical safety evaluations, well-designed clinical trials, long-term monitoring of recipients and post-market data collection should be developed [83, 84].

In addition, harmonization of international standards will promote greater collaboration among investigators, manufacturers, clinicians, advocacy groups and regulatory agencies and therefore help to expedite and ensure equitable access to stem cell-based therapies in pediatric medicine [84, 86].

## New technologies and innovation

### Genome editing techniques (CRISPR–Cas Systems) to correct genes in children

The development of various approaches to using CRISPR gene-editing technology in children with genetic disorders has opened new possibilities for treating these disorders. Previous efforts to treat a genetic disorder have used a modified virus in the child’s body to insert a “normal” copy of the gene into the child’s DNA. Although these innovations demonstrate a promise as a proof-of-concept; however, the majority of applications still remain research. For routine implementation for children; the long-term safety of use; durability of correction; reproducibility of manufacturing; and regulatory compliance must be demonstrated through clinical trials prior to use in a routine clinical setting. Although gene editing is seeing more use currently, both in early clinical trials and as a proof of concept, most of the applications included in this article remain investigational; therefore, they cannot yet be considered standard of care.

Using CRISPR/Cas systems, we can now make more intricate DNA edits for the child’s use, which can help reduce long-term medication costs to suppress the immune response to an organ transplant. CRISPR/Cas provides a new method for researchers to modify DNA, enabling the creation of cells from individuals with monogenic disorders, including metabolic leukodystrophies, immunodeficiencies, and congenital blood disorders.

Current preclinical studies are assessing whether high-fidelity CRISPR gene editing can be performed in patient-derived iPSC lines to generate haploidentical corrected progenitor cells, with the goal of reducing the risk of long-term infections and complications associated with solid organ transplantation [27, 28, 64]. Preclinical research demonstrates the viability of creating gene-edited neural and hematopoietic lineages that persistently engraft in experimental models. These data support the future application of genome correction strategies to augment current transplantation methods in limited pediatric indications; however, their clinical application remains investigational. Additional concerns related to off-target effects, incomplete editing, and potential genotoxicity will require extensive regulatory scrutiny and thorough safety assessment before implementation in pediatrics.

CRISPR-Cas9-based gene-editing therapies for sickle cell disease and transfusion-dependent b-thalassemia [94, 95]. represent examples of highly successful translational stem cell research. In these successful approaches, autologous (i.e. the patient’s own) hematopoietic stem cells are modified ex vivo to disrupt the BCL11A erythroid enhancer, resulting in restoration of fetal hemoglobin production and reduction of pathological effects of defective beta-globin synthesis [94, 96]. Many clinical trials have shown significant therapeutic benefit, such as elimination or reduction in vaso-occlusive crises experienced by patients with sickle cell disease, and achievement of transfusion independence for many patients with transfusion-dependent beta-thalassemia [95]. This outcome marks a significant milestone for regenerative medicine and provides an excellent example of successful translation from mechanistic discovery to clinical implementation [94]. Furthermore, these successful outcomes underscore the necessity for ongoing safety monitoring, standardization of manufacturing processes, regulatory oversight, and interdisciplinary collaboration in the ongoing advancement of next-generation stem cell therapy [95, 96].

### Organoids and 3D bioprinting for disease modeling and therapy

In addition, with the numerous advances in genome editing, researchers are now using organoid platforms to better model childhood diseases using 3D culture systems that resemble the structure and function of the original tissue. Organoids can be built from iPSCs or tissue-specific progenitor populations (e.g., liver organoids for the study of liver disease, brain organoids in the context of developing brain tumors, and heart organoids to study congenital heart disease) to better understand the underlying mechanisms of various diseases in children by evaluating drug responses and creating and optimizing of new therapies using cell-based approaches under controlled experimental conditions [32, 97, 98]. Over the last several years, organoid platforms have shifted from their initial use as observational models to experimental models with a specific aim of targeted manipulation. Integrating three-dimensional bioprinting technology has enabled researchers to create structurally based organoids that mimic the characteristics of a specific disease and therefore serve as investigational scaffolds for regenerative medicine and/or the repair of damaged/diseased tissues. For individuals with congenital anomalies, to date, there are experimental models that can support preclinical development of regenerative cardiac therapies using bioprinted MSC-based constructs. Early studies have shown that using bioprinted, MSC-enriched constructs can create a modestly improved ejection fraction, and better functional outcomes will occur with these types of tissues; although the utility of bioprinted cardiac tissues remains pending while awaiting additional studies conducted throughout the next several years [61]. Early in translation, organoids and bioengineered printed tissue have already begun to change the prospects of pediatric cell therapy.

The bioengineered and organoid based stem cell technologies are mostly used as experimental research tools, however these technologies have been very useful in modelling disease and developing personalized medicine. Translational issues constitute the main obstacle to broad therapeutic application.

### Artificial intelligence and computational modeling in stem cell prediction

As biological mechanisms become more complex, artificial intelligence is poised to be an ally in explaining stem cell function. Recent studies indicate that machine learning algorithms can accurately predict therapeutic potency, differentiation trajectories, and optimal donor or manufacturing environments [79]. In pediatric environments, where clinical time windows are limited and clinical deterioration may occur within hours, AI modeling will enable effective clinical decision-making about the product before its use in transplantation, mitigating clinical risk and reducing wait time. AI is already being fitted on datasets from MSC trials in cerebral palsy, neonatal hypoxic–ischemic encephalopathy, refractory rheumatic disease, and metachromatic leukodystrophy, which include predictive indices for response and safety signals over time [49, 53, 72]. Computational modeling is being applied to small biomarkers in MSC EV preparations, increasing their viability for cell-free treatment of diseases in the newborn period [29, 51, 75]. As modeling evolves, AI should move beyond being just an analytical tool to a dynamic design engine that drives clinical protocols, manufacturing processes, and approaches to long-term monitoring.

### Combination therapies and cell-free regenerative strategies

Ongoing innovation has expanded therapeutic options from conventional stem cell transplantation therapies to hybrid therapies that incorporate cellular agents with bioengineered or pharmacologic adjuncts. Cellular agents like MSCs have the broadest immunomodulatory properties and have been used in combination with conventional immunosuppressive therapies in pediatric patients with severe aplastic anemia, demonstrating hematologic improvements, recovery, and durable engraftment [99]. Similar combination therapies are being explored in cardiology, neurologic disorders, and refractory inflammatory conditions, enhancing the cell’s inherent reparative capacity while potentially decreasing cumulative systemic toxicity of alternative treatment. The path towards cell-free therapy, specifically MSC-derived EV nutrition, marks another valuable development in regenerative guidance. EVs contain a potpourri of components, including microRNAs, proteins, lipids, and metabolic mediators, and act like the parent cell without the complications of cellular engraftment and the uncontrolled risk of expansion. Preclinical studies and early-stage clinical investigations demonstrate the safety and tolerability of EV therapy as the sole treatment for conditions in the neonatal population, including lung disease, perinatal brain injury, and multiorgan inflammatory syndromes [51, 74–76]. Their controllability, stability, scalability, and limitations regarding immunoinflammatory activity make this treatment mode a good candidate for developmental and regulatory consideration in pediatrics. The combination of genome editing, organoid platforms, artificial intelligence, biologics, and vesicle therapies represents a suite of new technologies that will shape the future of pediatric regenerative medicine. Historically, early intervention trials were mainly limited to interventions in the context of transplantation and engraftment. Now, with the merging of genome editing and a capacity to manipulate biology itself, including bioengineering and the development of new regenerative therapies, the transition from potentially rehabilitative to potentially transformative therapies.

## Future perspectives and clinical translation

The future of pediatric stem cell therapy is becoming increasingly apparent as personalized, precise medicine gains wider acceptance. This means regenerative treatments will be tailored to each child’s specific genetic, immunologic, and developmental needs. Personalized approaches harness the ability to obtain patient-specific cells, including iPSCs, and to genetically modify them to develop the required cell types. These approaches assure minimal immune rejection and personalized therapeutic effectiveness for monogenic and complex pediatric diseases [28, 35]. Early clinical experience demonstrates that personalized rather than conventional stem cell therapies can markedly improve outcomes across a variety of conditions, from congenital hematologic abnormalities to neurodevelopmental syndromes [30, 50, 72]. Actualizing this vision will require a significant global collaboration and data-sharing efforts. Harmonized registries, standard outcome measures, and shared biobanks will provide cross-institutional opportunities for analyses that may hasten mechanistic understanding and therapy optimization [64, 100]. Pediatric regenerative medicine is highly complex and will require not only a robust data infrastructure but also cultural and ethical sensitivity, as well as collaboration across global networks, to ensure equity and comprehensive safety oversight [46, 49]. Mandating harmonization of regulatory processes remains a significant barrier for the international dissemination of pediatric stem cell therapy. For various reasons, countries have distinct regulatory frameworks, and ethical dilemmas arise when working with children. This poses a challenge for multi-center studies and the seamless implementation of new therapeutic modalities [53, 68]. Coordinated regulatory processes will need to support trial approval, support open data, and enable safety results in a timely and safe manner for regenerative therapies to be adopted for clinical practice. This will necessitate both mutual recognition agreements and shared recommendations for children [32, 101]. The field of regenerative pediatrics is poised for significant change in the near future. New technologies will enable greater accuracy, reproducibility, and mechanistic research, including gene editing, organoid platforms, exosome-based therapies, and artificial-intelligence-driven predictive modeling [51, 61, 74, 75, 79, 98, 102]. Additionally, combination strategies using cell-based therapies in conjunction with biomaterials, targeted small molecules, or immunomodulatory drugs will be available to increase therapeutic efficacy while minimizing potential side effects. All of these advancements foreshadow a future in which pediatric regenerative medicine is not only more accurate but also more scalable, ethically sound, and accessible to people around the world. In conclusion, the future of pediatric stem cell therapy will depend on personalized approaches, international collaboration, novel frameworks, and new technologies. All translational efforts will need to ensure strong oversight of safety and the ability to be flexible in adding new devices. The next decade may set the standard of care for children with regenerative, personalized, and easily accessible treatments.

## Conclusions

Over the past two decades, stem cell-based therapies have emerged as a promising area of pediatric regenerative medicine, demonstrating potential across a broad range of diseases. However, the evidence supporting different stem cell platforms varies considerably. Hematopoietic stem cell transplantation is an established therapy supported by extensive clinical experience and long-term outcome data, whereas mesenchymal stromal cells, extracellular vesicles, gene-edited products, organoids, and many iPSC-derived therapies remain investigational and require further clinical validation.

Despite significant advances in understanding stem cell biology, immunomodulation, paracrine signaling, and tissue repair, important challenges remain. Long-term safety, manufacturing standardization, reproducibility, potency assessment, regulatory approval, and ethical considerations—particularly in pediatric populations—continue to limit widespread clinical implementation. Future progress will depend on rigorous clinical trials, long-term monitoring, international collaboration, and the development of safe, effective, and equitable therapies.

Emerging technologies, including genome editing, organoid systems, extracellular vesicle therapeutics, 3D bioprinting, and artificial intelligence-assisted approaches, are expected to accelerate the field. Ultimately, the successful translation of pediatric stem cell therapies will require balancing innovation with safety, ethical responsibility, affordability, and individualized patient care.

## Data Availability

No datasets were generated or analysed during the current study.
